# Rational Design of Sustainable Liquid Microcapsules for Spontaneous Fragrance Encapsulation

**DOI:** 10.1002/anie.202110446

**Published:** 2021-09-17

**Authors:** Marianna Mamusa, Rosangela Mastrangelo, Tom Glen, Sergio Murgia, Gerardo Palazzo, Johan Smets, Piero Baglioni

**Affiliations:** ^1^ Dipartimento di Chimica “Ugo Schiff” Università degli Studi di Firenze & CSGI, (Consorzio per lo Sviluppo dei Sistemi a Grande Interfase) via della Lastruccia 3 Sesto Fiorentino (FI) I-50019 Italy; ^2^ School of Physics and Astronomy University of Edinburgh Edinburgh EH9 3FD UK; ^3^ Dipartimento di Scienze della Vita e dell'Ambiente Università degli Studi di Cagliari & CSGI, (Consorzio per lo Sviluppo dei Sistemi a Grande Interfase) via Ospedale 72 Cagliari 09124 Italy; ^4^ Dipartimento di Chimica Università di Bari “Aldo Moro” & CSGI, (Consorzio per lo Sviluppo dei Sistemi a Grande Interfase) Via Orabona 4 Bari I-70126 Italy; ^5^ The Procter & Gamble Company Temselaan 100 1853 Strombeek Bever Belgium

**Keywords:** fragrance, microencapsulation, microplastic, Raman microspectroscopy, spontaneous phenomena

## Abstract

The high volatility, water‐immiscibility, and light/oxygen‐sensitivity of most aroma compounds represent a challenge to their incorporation in liquid consumer products. Current encapsulation methods entail the use of petroleum‐based materials, initiators, and crosslinkers as well as mixing, heating, and purification steps. Hence, more efficient and eco‐friendly approaches to encapsulation must be sought. Herein, we propose a simple method by making use of a pre‐formed amphiphilic polymer and employing the Hansen Solubility Parameters approach to determine which fragrances could be encapsulated by spontaneous coacervation in water. The coacervates do not precipitate as solids but they remain suspended as colloidally stable liquid microcapsules, as demonstrated by fluorescence correlation spectroscopy. The effective encapsulation of fragrance is proven through confocal Raman spectroscopy, while the structure of the capsules is investigated by means of cryo FIB/SEM, confocal laser scanning microscopy, and small‐angle X‐ray scattering.

## Graphical abstract

The use of a PEG‐*g*‐PVAc amphiphilic graft copolymer together with hydrophobic fragrances in aqueous medium leads to the spontaneous formation of colloidally stable suspensions of liquid coacervate droplets. Arrows indicate the local turbulences sustaining the spontaneous mixing of the components. The bottom panel shows the confocal Raman microspectroscopy mapping of a multicompartment coacervate droplet with carvone as fragrance.

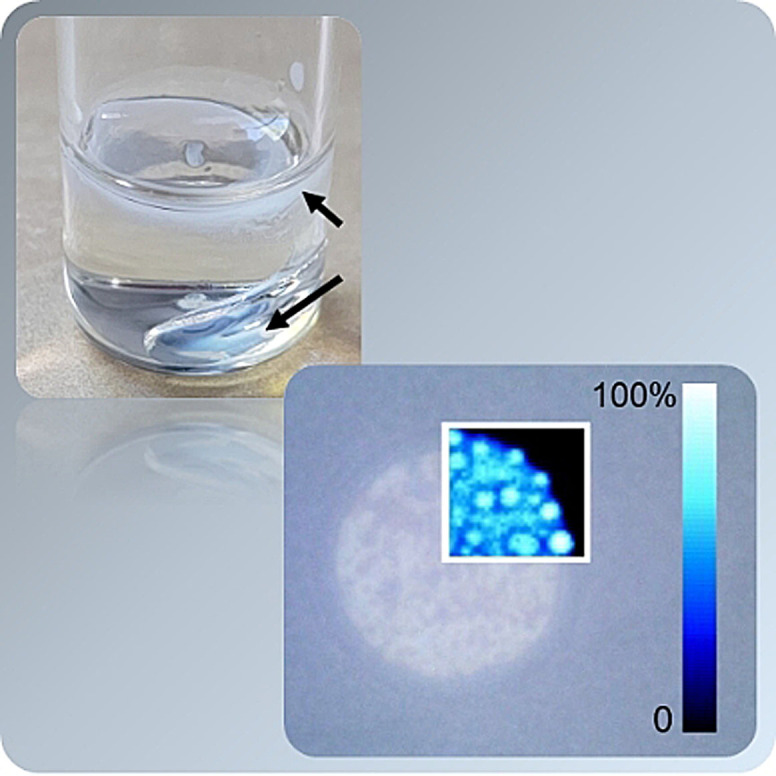

## Introduction

Encapsulation entails the enclosure and protection of an active compound against degradation, uncontrolled diffusion and, ultimately, loss. At present, encapsulation methods are widely used in pharmaceutical, chemical, cosmetic, food, and printing industries.[^1^, ^2^] In food formulations, for instance, flavors are encapsulated to avoid degradation or migration through the food matrix;^[3]^ drugs, on the other hand, can be included in microcapsules to improve their bioavailability, taste, or odor.^[4]^ Encapsulation also ensures the stability of the final products, thereby extending their shelf‐life.

Microcapsules are usually defined as micron‐sized particles, or droplets, included in a solid shell.^[5]^ The shell material ensures the protection and transport of active compounds until external stimuli trigger the release. The addition of scents to formulations is a challenging task: the high volatility of most perfume raw materials, their immiscibility with water, and their sensitivity to light and oxygen can lead to discoloration and the development of undesired scent notes. Numerous research efforts have attempted to tackle this issue by focusing on the microencapsulation of fragrances with various materials, most polymeric in nature.[^6^, ^7^]

To this purpose, aminoplast resins, such as the melamine–formaldehyde type,^[8]^ show the best performances in the field of fragrance encapsulation. Although cheap and easy to synthesize, these materials suffer from a lack of biodegradability, especially when leaking via wastewaters.^[9]^ Microcapsules for fragrance encapsulation should also grant a sustained release: in laundry products, for example, an efficient scent deposition on fabrics is perceived as freshness, resulting in a lower number of washing cycles. This is crucial to reduce the major source of microplastic pollution, i.e., microfibers from home washings of synthetic clothing: they account for 35 % of the total microplastic burden in the oceans (other significant sources are tire erosion, 28.3 %, and city dust, 24.2 %).[^10^, ^11^]

In light of the previous arguments, the fragrance technological sector is continuously in search of new, more efficient encapsulation routes with a lower environmental impact, especially considering the substantial societal impact of the home and laundry care field, whose market revenue is estimated in more than US$ 50 billion worldwide.^[12]^ Examples of possible replacements are low‐formaldehyde microcapsules obtained via the use of scavengers,^[13]^ or systems based on natural materials such as chitosan.^[14]^

A common traditional method to obtain core–shell microcapsules is emulsion polymerization, in which a monomer feed is dissolved in a water‐immiscible oil, and the polymerization process takes place at the liquid–liquid interface, giving rise to water‐dispersed capsules containing the oil.[^15^, ^16^] By carefully choosing the wall material, this technique can yield microcapsules with good colloidal stability, stimuli‐responsiveness, and high diffusion barrier properties.[^17^, ^18^] Encapsulation processes via in situ polymerization, however, frequently require the use of cosolvents and emulsifiers, as well as polymerization initiators and crosslinkers to obtain the desired product, in addition to the necessity of energy input (mixing, heating) and purification steps. In other words, their environmental profile is poor. Nano‐ and microcapsules can be prepared via solvent displacement (sometimes referred to as nanoprecipitation, solvent diffusion, or interfacial deposition), a simple method making use of preformed polymers and requiring little to no energy input.[^19^, ^20^, ^21^] Briefly, the polymer (and possibly a drug or an active chemical, as needed) is dissolved in a water‐miscible solvent, and then such mixture is added to water to trigger precipitation; the spontaneous formation of polymer nanoparticles occurs in conditions of supersaturation, via either a nucleation‐and‐growth or a nucleation‐and‐aggregation mechanism.[^22^, ^23^] A variant in which the solvent is only partially miscible with water is named emulsion–diffusion.^[24]^

In 2005, Ganachaud and Katz reviewed a series of papers describing spontaneous polymer precipitation and reinterpreted the findings from the point of view of the polymeric Ouzo effect.^[25]^ This is originally described as a nonequilibrium, spontaneous emulsification process originating from homogeneous liquid–liquid nucleation in a metastable region of a ternary phase diagram, between the binodal and the spinodal curves.^[26]^ Under this new light, “solvent displacement process,” “coacervation by addition of a nonsolvent,” “nanoprecipitation,” and “spontaneous emulsification” appear as different terminology describing the same physical phenomenon, which can and has been used to obtain polymeric nano‐ and microparticles filled with drugs, oils, or other payloads.^[27]^ In the presence of additives pre‐mixed with the polymer, the Ouzo region can shift in the phase diagram due to the change of polymer solubility in water imparted by the additive molecules.^[28]^ Moreover, the extension of the Ouzo region can be sensitive to the kinetics of mixing (e.g. slow or fast addition of one solution into the other).^[23]^

Most studies present in the literature propose the use of additives, mixtures of different polymers, or cosolvents.^[29]^ Also, the use of polymers presenting a high *T_g_*, such as PMMA, leads to the precipitation of solid nano‐ or microcapsules: this is usually desired; however, it entails several steps for the correct separation of the particles from the reaction medium to be utilized in the final formulation, and might require surface functionalization or formulation pH adjustment to ensure colloidal stability. In this work, we employ an amphiphilic nonionic block copolymer of poly(ethylene glycol)‐*graft*‐poly(vinyl acetate), PEG‐*g*‐PVAc, in the presence of a fragrant compound, or a mixture thereof, and water. Mixed in the appropriate ratios, the three components give rise spontaneously to liquid microspheres characterized by excellent colloidal stability, without the need for additional thickeners to modify the medium viscosity. The copolymer's *T_g_* is below 0 °C,^[30]^ which allows for polymer flexibility at room temperature and thermodynamically driven self‐assembly,^[31]^ while the biodegradable polymer blocks[^32^, ^33^, ^34^] make PEG‐*g*‐PVAc an ideal candidate to replace other less sustainable encapsulation systems. The microcapsules’ formation process appears similar to the Ouzo effect, except for the fact that the miscibility of the organic solvent (i.e., the fragrance) with water is extremely low. The key factor is the affinity of the fragrance for the polymer, which can be expressed in terms of Hansen Solubility Parameters (HSP).^[35]^ HSP derive from Hildebrand's total solubility parameter,^[36]^ divided by Hansen into its three partial components [Eq. 1].

(1) $$\delta_{tot}=\sqrt{\delta_{D}^{2}+\delta_{P}^{2}+\delta_{H}^{2}}$$

These refer, respectively, to the dispersion forces (*δ*_D_), polar interactions (*δ*_P_), and hydrogen bonding (*δ*_H_) contributions to the cohesive energy density. They are, therefore, representative of the molecular properties of a certain compound; the closer the HSP of two substances, the higher the affinity between them. This approach can help identify substances, and mixtures thereof, that can be encapsulated by a single polymer.^[37]^ The systems are characterized by means of microscopy techniques (optical, cryo FIB/SEM, and confocal Raman), as well as fluorescence correlation spectroscopy and small‐angle X‐ray scattering.

## Results and Discussion

**Figure 1:**
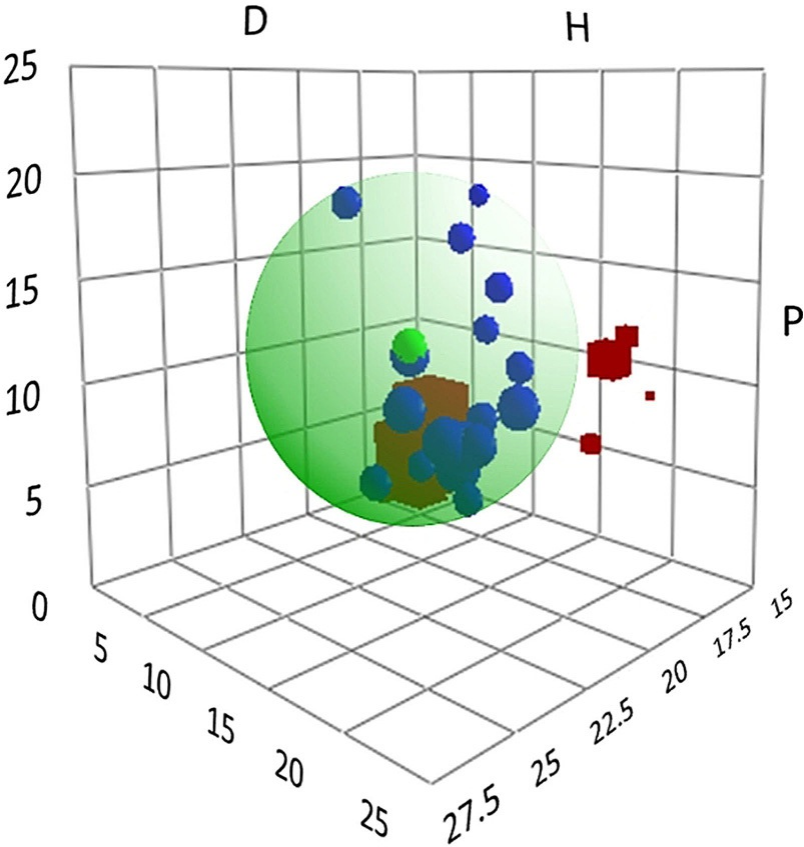
Hansen solubility sphere calculated for PEG‐*g*‐PVAc. Each axis corresponds to one of the three HSP: D=Dispersion forces parameter, P=Polar interactions parameter, H=Hydrogen bonding parameter. The blue spheres represent the good solvents, while the red cubes represent the bad solvents. The small green sphere represents the center coordinates, corresponding to the HSP of the polymer.

The HSP of PEG‐*g*‐PVAc were obtained experimentally by performing a set of solubility tests of the polymer in different solvents, including common fragrant molecules (Table S1, Supporting Information). Based on Equation S1 (see the Supporting Information), we calculated the Hansen sphere, shown in Figure 1, encompassing all the good solvents, i.e., those solvents having a high affinity with the polymer. The sphere origin corresponds to the HSP of the copolymer: *δ*_D_=18.9, *δ*_P_=10.4, *δ*_H_=6.9. It is worth mentioning that the data were also treated with the Double Sphere algorithm by Yamamoto,^[38]^ which at a first glance might seem most appropriate for an amphiphilic diblock copolymer. However, calculations yielded two interpenetrating spheres, none of which matched the respective HSP values of the PEG and PVAc single blocks nor did provide any improvement in fit accuracy—in fact, the best fit included two bad solvents within the spheres. Since the driving force for encapsulation, here, seems to be the affinity of the fragrance molecules for *both* polymer blocks, the classic sphere representation of the Hansen solubility space appears suitable for the present scope. HSP can be used to choose appropriate mixtures of perfume raw materials according to their compatibility with the carrier polymer, but also to replace possibly toxic or allergenic compounds with more benign ones having similar chemistry.^[39]^

**Figure 2:**
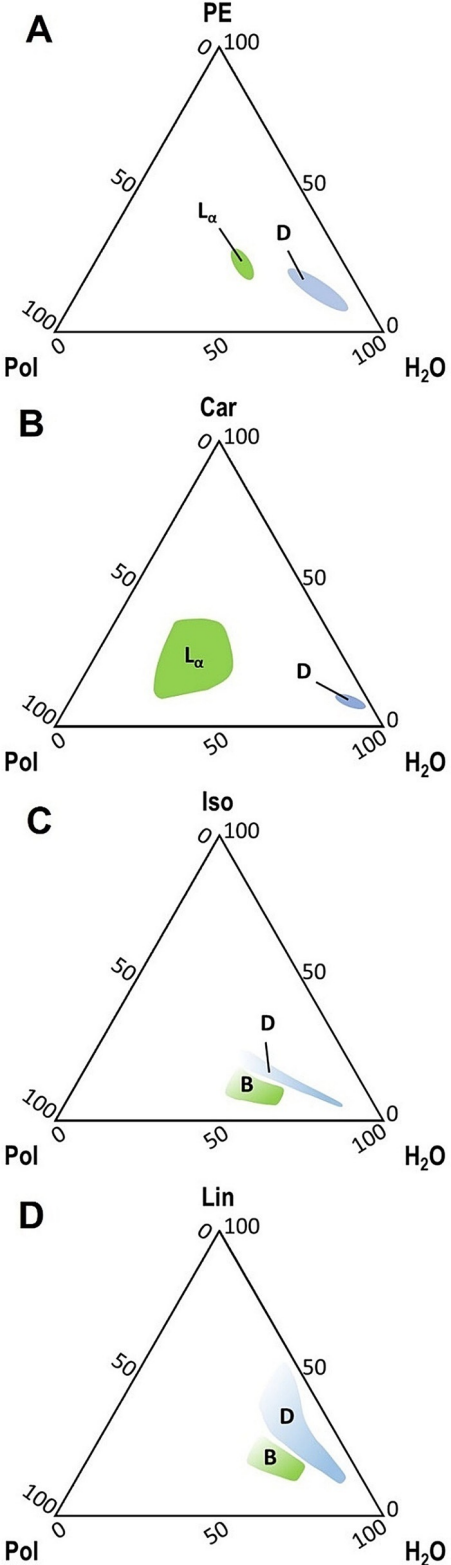
Partial Gibbs phase diagrams of the PEG‐*g*‐PVAc/perfume/water systems at 25 °C; “Pol”=PEG‐*g*‐PVAc. A) 2‐phenyl ethanol (PE); B) l‐carvone (Car); C) isoeugenol (Iso); D) linalool (Lin). “D” indicates the droplet region, “L_α_” indicates a lamellar mesophase, and “B” indicates a generic gel‐like birefringent phase. Concentrations on the axes are wt %.

We will focus on four common perfume raw materials (PRMs), all classed as good solvents for our polymer and poorly soluble in water (properties shown in Table 1): 2‐phenyl ethanol (PE), l‐carvone (Car), linalool (Lin), and isoeugenol (Iso). Figure 2 shows the four partial phase diagrams of the PEG‐*g*‐PVAc/perfume/water systems, evidencing regions of relative concentrations where the three components form remarkably stable dispersions of micron‐sized droplets (“D” regions). We verified that no droplet region exists in those systems where a bad solvent is present, such as α‐pinene or undecenyl aldehyde, or a water‐miscible good solvent, such as acetone. The complete phase diagrams for PE and Car have been discussed in detail in previous work.^[40]^ The “D” region for Lin is remarkably extended in comparison with the other three PRMs; this does not seem to correlate with any of the partial HSP, but could be explained by the flexibility of the terpenoid molecule, allowing it to intermingle in the polymer structures.

**Table 1:** HSP and molecular structures for the compounds discussed in this work (HSP units are MPa^1/2^). Solubility in water is given in g L^−1^ for the fragrance compounds.

Material	*δ*_D_	*δ*_P_	*δ*_H_	Solub. H_2_O	Molecular structure
PEG‐*g*‐PVAc	18.9	10.4	6.9	–	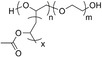
2‐phenyl ethanol	18.3	5.6	11.2	22	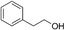
l‐carvone	17.5	5.8	3.7	1.3	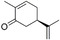
linalool	16.8	2.9	6.9	1.5	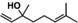
isoeugenol	18.9	5.7	9.8	0.8	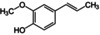
α‐pinene	16.9	1.8	3.1	0.002	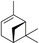
anisaldehyde	19.4	11.9	8.3	4.3	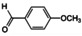

In each phase diagram, the droplet regions are flanked by areas of birefringent liquid crystals at higher polymer content, which were identified as lamellar mesophases (“L_α_”) in the PE and Car systems.^[40]^

**Figure 3:**
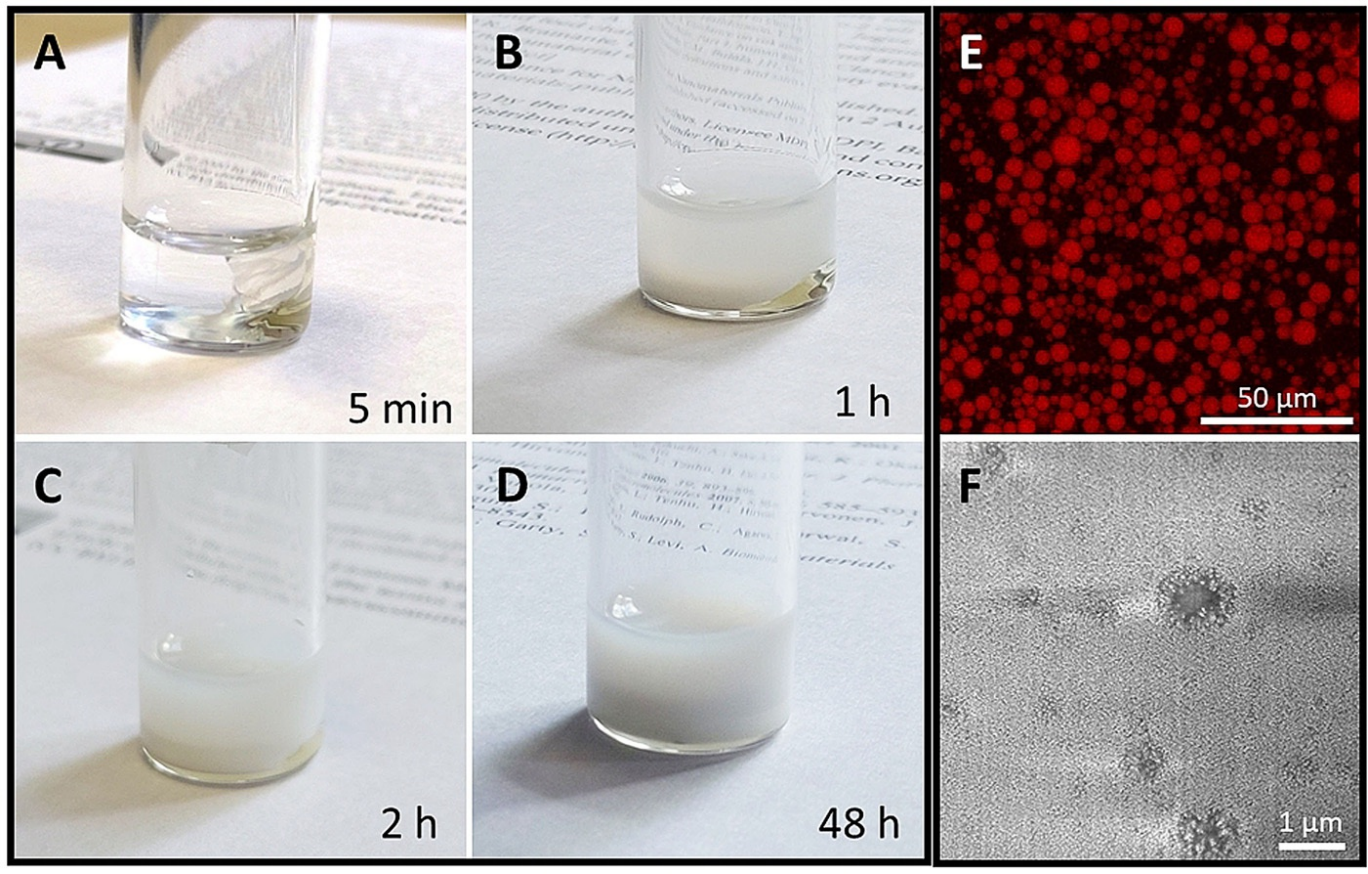
A–D) Temporal sequence of spontaneous formation of PEG‐*g*‐PVAc/PE/water liquid microcapsules (see also the movie provided as Supporting Information). E) CLSM image of the final sample, prepared with rhodamine B‐labeled polymer. F) Cryo FIB/SEM image of the final sample.

The samples pertaining to the “droplet” regions appeared milky and did not show any macroscopic phase separation. As anticipated, the formation of these particles was spontaneous: regardless of the order of mixing of the components, the same PEG‐*g*‐PVAc/PE/water droplets formed if the correct ratios between the three components were respected. As we show in the video in the Supporting Information, the process can occur spontaneously without mixing or heating the system, driven solely by molecular diffusion sustained by concentration gradients across the sample. Figure 3 A–D summarizes the temporal sequence of dispersion formation, which appears complete after 48 h; confocal laser scanning microscopy (CLSM; Figure 3 E) imaging of the final sample, prepared with fluorescently labeled polymer, shows an apparently homogeneous size distribution of the particles.

In fact, cryo FIB/SEM images revealed the presence of submicron particles, which are below the size resolution of optical microscopes: in Figure 3 F, micron‐ and submicron‐sized structures are evident, which present a clear micelle‐like aspect with a solid core surrounded by a fuzzy corona. When observed with a microscope, the droplets appeared to bump into each other frequently without coalescing: this behavior demonstrates an outstanding elasticity of the interfacial polymer film, which explains the long‐term stability of at least 6 months of these droplets. Dilution with further water caused the droplets to burst as the border of the single‐phase region was crossed, while a typical sample taken well inside the droplet regions could withstand freezing at −18 °C, heating up to 70 °C, and centrifugation at 5000×g without the occurrence of phase separation.

**Figure 4:**
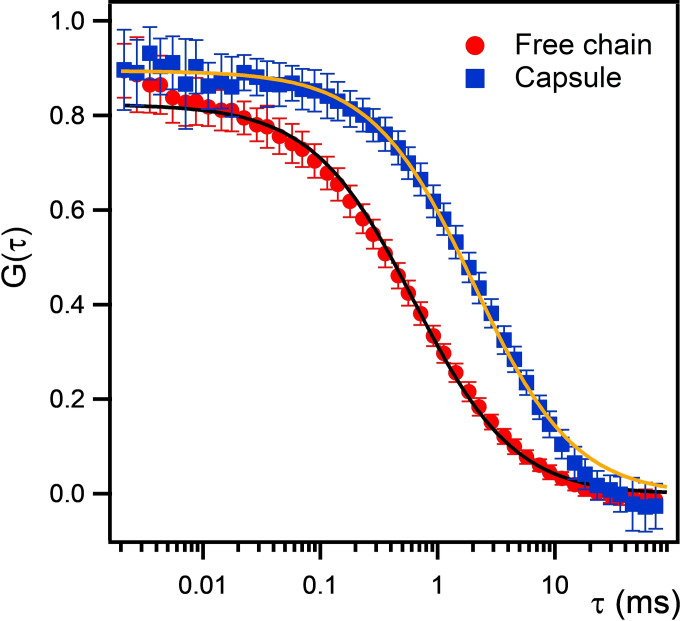
FCS autocorrelation curves of rhodamine B‐labeled PEG‐*g*‐PVAc in water (red circles) and in microcapsules containing 2‐phenyl ethanol (blue squares). Solid lines are model fits to Equation S2.

The liquid state of the microcapsules was assessed by means of fluorescence correlation spectroscopy (FCS), using the PEG‐*g*‐PVAc/PE/water system as representative. FCS decay curves, shown in Figure 4, were analyzed according to Equation S2 (Supporting Information), while the diffusion coefficients of the labeled species were obtained through Equation S3.

The measured diffusion coefficient of the rhodamine B‐labeled PEG‐*g*‐PVAc in dilute aqueous solution was 2×10^−11^ m^2^ s^−1^. Then, microcapsules were prepared with the fluorescently labeled polymer and PE in water, and the FCS curves were acquired for a region inside the capsule. In this case, the measured diffusion coefficient was 6×10^−12^ m^2^ s^−1^, suggesting that the polymer chains were diffusing in a viscous fluid, i.e., that some mobility was retained in the core of the polymer capsules. NMR diffusion experiments, performed on the whole sample, returned diffusion coefficients for the PEG‐*g*‐PVAc of the same order of magnitude of that measured by FCS within the droplets. This confirms that the NMR peak (observable only in the liquid state) of the polymer was mainly due to the PEG‐*g*‐PVAc in the liquid state within the capsules.

**Figure 5:**
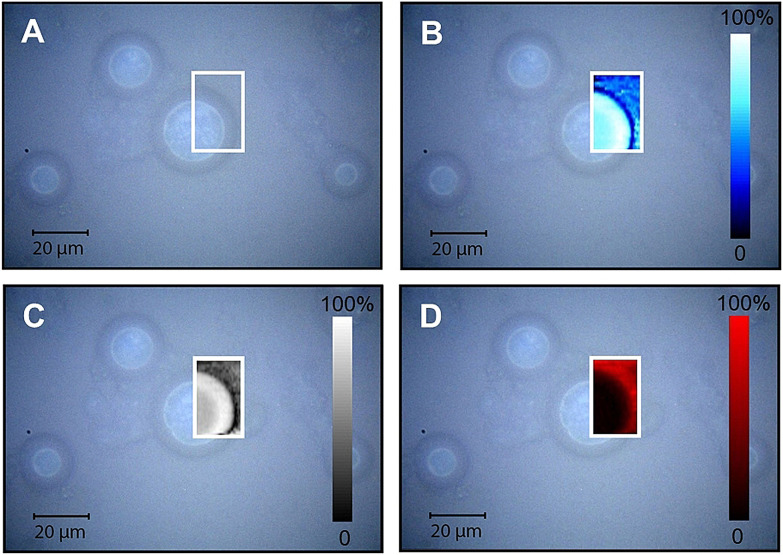
A) Optical image and B–D) confocal Raman mapping of capsules in the PEG‐*g*‐PVAc/Car/water system (see Figure S5, Supporting Information, for a representative spectrum): B) map of the intensity of the signal at 1644 cm^−1^ (C=C stretch); C) map of the intensity of the signal at 2930 cm^−1^ (C−H stretch); D) map of the signal‐to‐baseline ratio of the band centered at 3500 cm^−1^ (O−H stretch). Color bars represent normalized signal intensities.

The presence of the fragrances inside the polymer microcapsules was confirmed by means of confocal Raman microscopy. Figure 5 displays representative Raman maps, superimposed to the optical micrographs, of PEG‐*g*‐PVAc/Car/water capsules: a core–shell structure is evident, corroborating the results of CLSM imaging presented in our earlier work.^[40]^ The Raman maps of the C−H (Figure 5 B) and C=C (Figure 5 C) stretch signals present the highest intensities in the capsule shell. It must be noted that the Raman spectra recorded at different positions in the capsules presented identical signal patterns, almost superimposable to the pure perfume spectra (provided in the Supporting Information), suggesting that the polymer contribution to the C−H mapping was very low. Since the samples prepared with fluorescently labeled polymer exhibited ring‐like structures,^[40]^ we can conclude that the polymer constitutes the shell only, while Car fills the core but also penetrates the shell. The O−H stretch signal, as shown in Figure 5 D, was found only outside the capsule and, although faintly, at the capsule/bulk interface, revealing that water penetration is limited to the hydration of the PEG outer rim.

**Figure 6:**
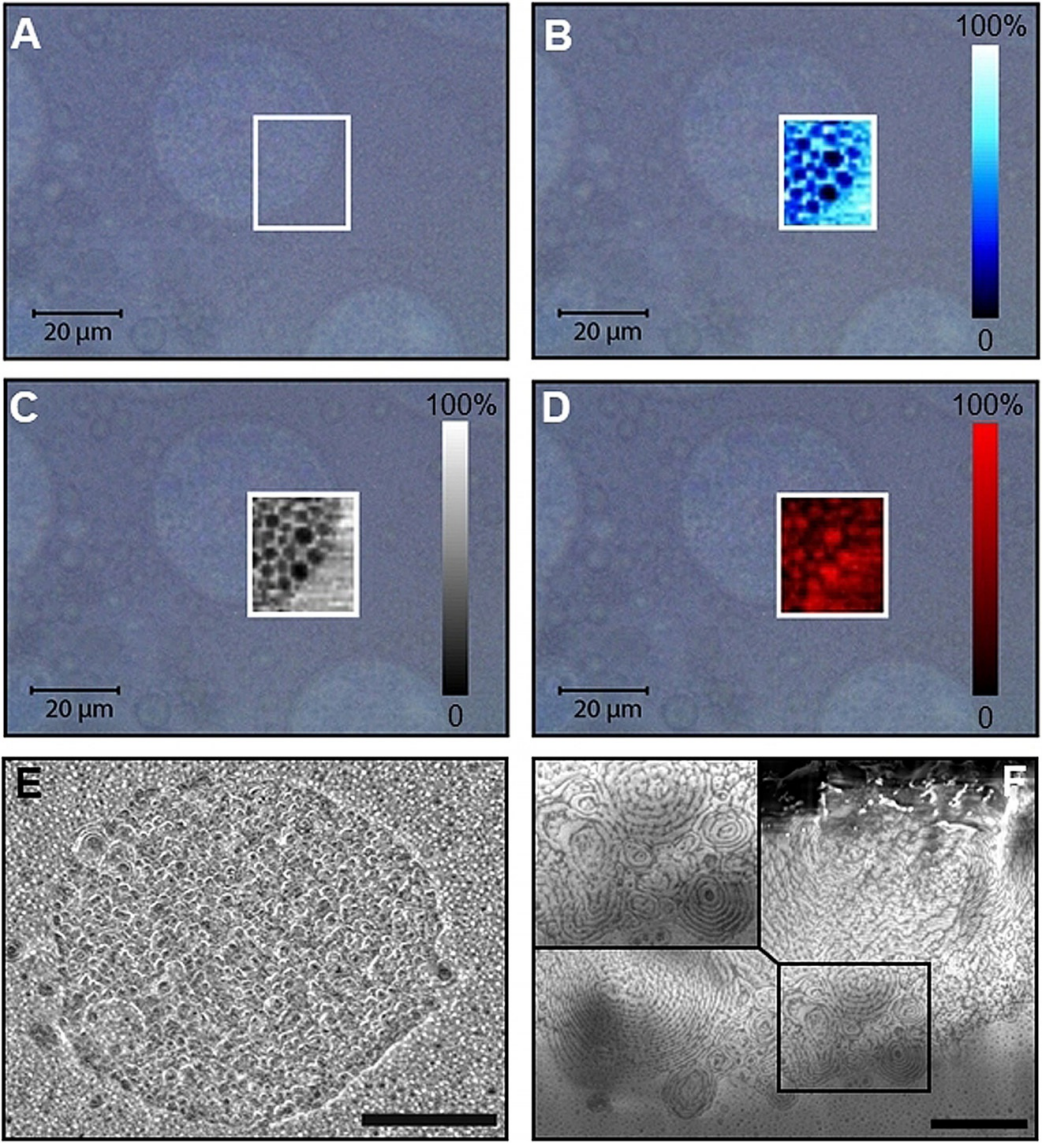
A) Optical image and B–D) confocal Raman mapping of capsules in the PEG‐*g*‐PVAc/Lin/water system (see Figure S6, Supporting Information, for a representative spectrum): B) map of the intensity of the signal at 1674 cm^−1^ (C=C stretch); C) map of the intensity of the signal at 2915 cm^−1^ (C−H stretch); D) map of the signal‐to‐baseline ratio of the band centered at 3450 cm^−1^ (O−H stretch); color bars represent normalized signal intensities. E,F) Cryo FIB/SEM of the same sample (scale bar: 1 μm); the inset shows some of the onion‐like structures in more detail.

In contrast, the system based on Lin (Figure 6) presented coexisting small (ca. 5 μm) and large (ca. 40–50 μm) capsules, the latter showcasing a multicompartment structure. The maximum intensity of the C−H and C=C stretch bands (Figure 6 B,C) was found in the scaffold, while that of the O−H band (originating primarily from H_2_O, Figure 6 D) was found inside the “holes”, suggesting a water‐in‐oil type of structure. The C−H and C=C signals were also mapped in the areas surrounding the capsules: this could be an artefact originating from the movement of smaller objects during 2D mapping, or it could be evidence that a dilute polymer‐in‐perfume solution constitutes the bulk phase of this dispersion. Pieces of evidence in favor of the latter hypothesis are the NMR diffusion results reported in the Supporting Information. By comparing the diffusivities of the perfumes in aqueous solution and in the formulations, it is possible to estimate the concentration of the fragrances not encapsulated (listed in Table S4). In the cases of PE (24 g L^−1^) and Car (4.7 g L^−1^), these values were close to their solubilities in water, while in the case of Lin, the concentration estimated by NMR diffusion measurements (29 g L^−1^) was twenty times larger. This suggests a non‐negligible presence of some complex between Lin and PEG‐*g*‐PVAc in the aqueous bulk.

Once again, cryo FIB/SEM was able to evidence structures that were not resolvable via the optical techniques: Figure 6 E,F reveals that the inner nanostructure of these objects is bicontinuous and even lamellar‐like, as suggested by the presence of onion‐like arrangements. A more quantitative description of the characteristic lengths in these structures was obtained by measuring the interlamellar distances along radial directions of the onion‐like objects (see Figure S9, Supporting Information). The most recurrent values ranged between 17 and 35 nm.

Small‐angle X‐ray scattering (SAXS) data, acquired on the same sample, are shown in Figure S10. The curve was fitted according to a core–shell model (see Supporting Information): the fit results (Table S2) describe particles with a 50 nm diameter, in agreement with the multicompartment structure shown in Figure 6 E.

As mentioned above, in systems containing Lin and Iso, the droplet areas in the phase diagrams were flanked by areas of gel‐like, birefringent structures (“B” in Figure 2). Some representative samples of these regions were observed in polarized light and probed through SAXS measurements. Optical micrographs (Figure S11) and SAXS curves (Figure S12) of samples containing Lin or Iso indicate a complex scenario: the systems are probably a mixture of different phases, as polarized light evidenced areas of weak local order, while the presence of structural defects at the nanoscale made characteristic SAXS peaks not clearly evident but partially superimposed to a low‐Q broad peak; the latter can be probably related to the presence of multicompartment polymer capsules (Figure S13). A clearer picture was provided by rheology measurements: according to Mezzenga et al.,^[41]^ the profiles of the storage (G′) and loss (G′′) moduli can be used to identify a specific ordered phase: G′>G′′, with G′ almost independent of frequency, (*ω*), indicates lamellar order; conversely, a strong dependence on *ω*, with G′′>G′ at low *ω* and a crossover occurring at higher *ω*, is characteristic of hexagonal phases. This approach is valid also for polymer‐based systems.^[42]^ The comparison of our data with characteristic rheological profiles of different phases led us to classify the Lin sample as preferentially lamellar and the Iso one as preferentially hexagonal (Figure S14). With this new evidence, we were able to hypothesize the position of characteristic SAXS peaks (Figure S12): in the Lin sample, the first peak at *Q*≈0.023 Å^−1^ would correspond to an interlamellar spacing of 27 nm; such value is in agreement with interlamellar spacings observed in the onion‐like objects in Figure 6 F and with previous data obtained on lamellar samples of the same polymer at similar compositions.^[36]^ By increasing the polymer:perfume ratio at a constant water content (55 % w/w), the samples showed more intense correlation peaks which could be more easily related to the prevalence of lamellar order (1:2 Q‐sequence) in the system. In both Lin and Iso samples, characteristic peaks shifted to higher Q values, suggesting a shrinking of interlamellar spacing or cylinder radius when the perfume content decreased (Figure S15).^[43]^

As mentioned above, the broad peak in the Iso and Lin SAXS curves can be related to the coexistence of spherical objects (likely those observed in multicompartment capsules) with structured areas. Upon dilution in water, at a constant polymer:perfume ratio, the peak shifted slightly to lower Q values in both samples (Figure S16); this probably indicates the swelling of capsules. Structural peaks, in contrast, became less intense when the water content increased, due to a partial disruption of the local order. SAXS data suggest that these polymer capsules are very stable, in agreement with their presence in wide areas of the phase diagrams, regardless of whether they exist alone or coexist with ordered structures. Furthermore, the role of the lamellar phase in the Lin samples could be similar to that described by Alexandridis et al. concerning the stabilization of emulsions:^[44]^ when the emulsification path crosses a region of L_α_ gel, the resulting nanoemulsion droplets are particularly homogeneous and stable owing to the ability of the lamellar microstructure to retain large quantities of oil.

The capsules containing Iso presented some technical difficulties for optical imaging and Raman mapping; therefore, we only provide CLSM images of the samples prepared with fluorescent PEG‐*g*‐PVAc (Figure S8). Here, the coacervate droplets exhibit a heterogeneous size distribution, and the red background suggests the existence of submicron particles in a similar way as that observed in other systems. These samples were stable against phase separation for at least 6 months.

**Figure 7:**
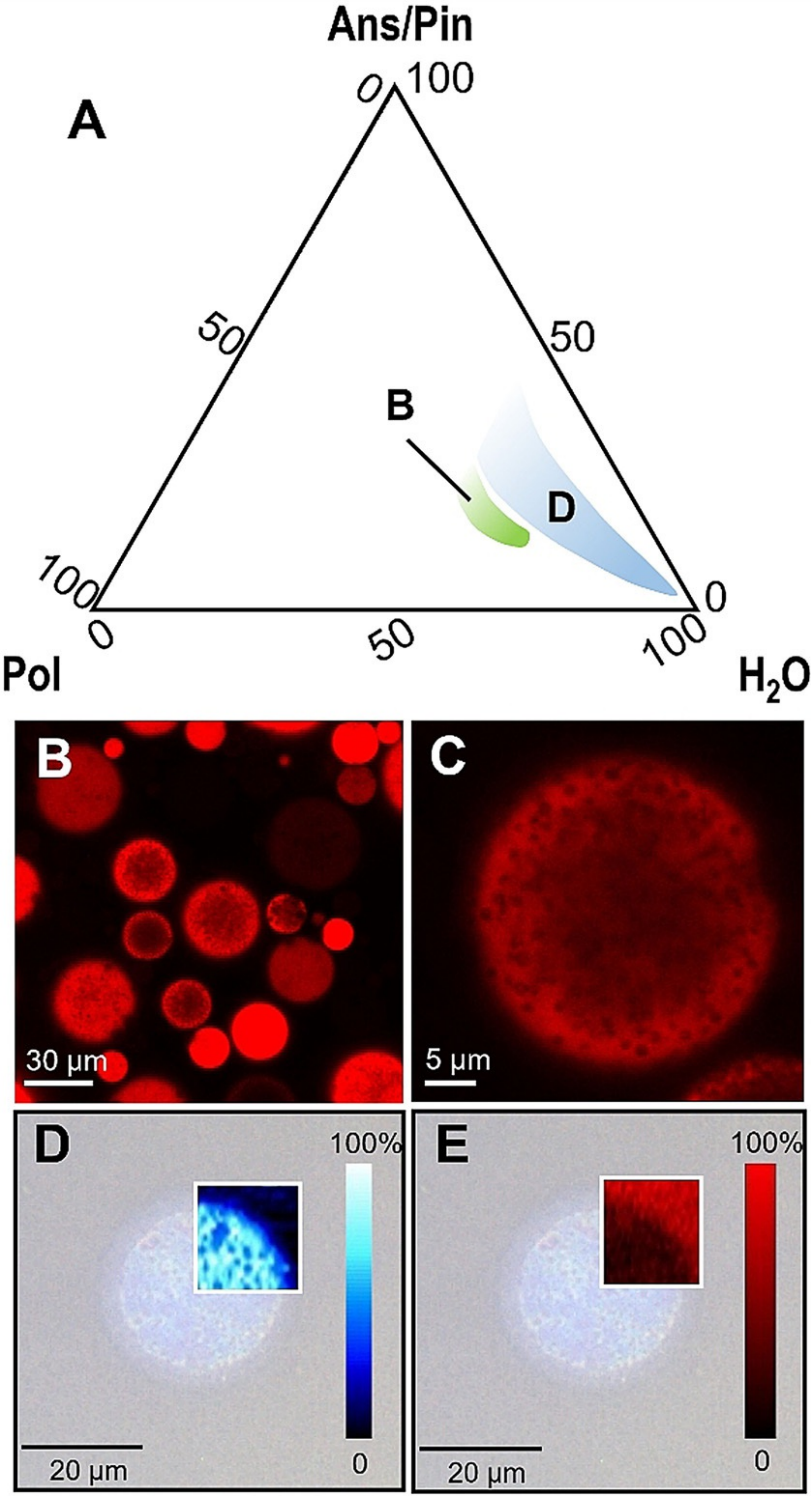
A) Partial Gibbs phase diagram at 25 °C of the PEG‐*g*‐PVAc/perfume/water system where perfume=83 % anisaldehyde (Ans)/17 % α‐pinene (Pin); “Pol”=PEG‐*g*‐PVAc. “D” indicates the droplet region, and “B” indicates a region of birefringent gel‐like phase. Concentrations on the axes are wt %. B,C) CLSM imaging of droplets in the PEG‐*g*‐PVAc/(Ans/Pin)/water system; the red signal is due to the rhodamine B‐labeled polymer. D,E) Raman mapping of the same capsules (see Figure S7, Supporting Information, for a representative spectrum): D) map of the intensity of the signal at 1600 cm^−1^ (C=O stretch); E) map of the signal‐to‐baseline ratio of the band centered at 3450 cm^−1^ (O−H stretch); color bars represent normalized signal intensities.

Finally, we applied the HSP approach to engineer a PRM mixture capable of forming capsules with our polymer. As proof of concept, we took into consideration α‐pinene (Pin), which does not form any droplet phases by itself in the ternary system with PEG‐*g*‐PVAc and water, as verified in previous work.^[40]^ Pin was identified as a bad solvent for PEG‐*g*‐PVAc, meaning that it lies outside its solubility sphere in the Hansen space. Here, the goal was to “bring” the desired solvent into the polymer's sphere by finding an appropriate mixture with a better solvent. Since the HSP of a mixture of solvents are a linear combination of those of the individual compounds, we can easily calculate that, for example, a 17/83 % mixture of Pin and anisaldehyde (Ans, see Table 1) will have HSP=(18.9, 10.1, 7.2), i.e., values very close to those of the polymer, and will be within the radius of the sphere. As predicted, the pseudo‐ternary diagram of the system PEG‐*g*‐PVAc/(Ans/Pin)/water (Figure 7) presented a wide region of droplet‐like aggregates, flanked by the usual area of birefringent gel‐like phase. CLSM imaging showed large capsules (20–40 μm) containing submicron‐sized non‐fluorescent droplets or voids. Raman mapping of the aldehyde C=O stretch band intensity (Figure 7 D) indicated the presence of perfume inside the coacervate matrix, except in such voids where there appeared to be water instead (Figure 7 E, mapping of the O−H band signal‐to‐baseline ratio). Unfortunately, the spectrum of Ans completely overwhelmed that of Pin, so that it was not possible to map the two compounds individually. Finally, the characterization of the B region via SAXS, optical microscopy, and rheology revealed the presence of a disordered lamellar phase very similar to the one observed in the Iso samples (see Figures S11, S12, and S14 in the Supporting Information).

## Conclusion

The encapsulation methods currently employed in several technological fields involve the use of solid‐like materials as constituents of the capsule shells: these prevent the deterioration or leaking of the active compounds. Nonetheless, an effective and sustained active release under certain external stimuli is also crucial. In laundry products, for example, the effective scent deposition on fabrics endows these with a feeling of freshness and cleanliness which allows to reduce the number of washing cycles. This results in a lower environmental impact, as microfibers from synthetic clothes are a major concern in terms of microplastic pollution in the oceans.

Moreover, solid‐shell microcapsules suffer from a lack of biodegradability when leaked into wastewaters. In this light, the technological sector of perfumes encapsulation is in strong need of more sustainable materials versus the currently employed systems, characterized by poor environmental profiles and sometimes inefficient fabrication processes,^[9]^ especially considering the pervasive use of home and laundry care products worldwide (estimated market revenue over US$ 50 billion).^[12]^ Nonionic polymers displaying a lower critical solution temperature behavior have been used for the encapsulation of poorly water‐miscible oils by coacervation for a long time,^[45]^ and in this work, we have reiterated such potential for a PEG‐*g*‐PVAc amphiphilic copolymer^[40]^ where the constituting blocks are biocompatible and biodegradable.[^32^, ^33^, ^34^] The novelty of this work is that, to the best of our knowledge, a rational design of such simple systems based on the HSP has never been presented. In recent work,^[46]^ Taden et al. have performed a systematic investigation on the formation of nanocapsules by interfacial polymerization of an acrylic copolymer, choosing the encapsulated terpenes by their HSP according to a criterion of poor compatibility with the shell; this led to capsules endowed with high diffusion barrier properties, which is a desirable characteristic when the fragrance release can be triggered following precise stimuli (in this case, pH variation). Herein, we have followed the HPS approach but according to an opposite strategy: we used a pre‐formed amphiphilic polymer in order to avoid the use of reaction initiators and the necessity of purification steps, and we used the HSP approach to establish which fragrances, or mixtures thereof, could be encapsulated by direct, spontaneous coacervation in water. The encapsulation was demonstrated by means of confocal Raman spectroscopy. Due to the low *T_g_* of the polymer, the coacervates did not precipitate as solids, but they remained suspended as liquid microcapsules, as proven through FCS, cryo FIB/SEM, and SAXS measurements. The barrier effect to the diffusion of the fragrances into the aqueous bulk is clearly limited by the water solubility, although low, of the terpenoid molecules; however, this property is also necessary to ensure the release of the perfume for the final application in liquid consumer products. In fact, for this kind of capsules, a burst release is readily triggered via dilution of the systems, leading to a complete destruction of the capsules as the border of the stability regions in the phase diagrams is crossed. However, other external stimuli, such as variations in local temperature, could be used in analogous systems.

The straightforwardness of preparation as well as the extraordinary colloidal stability and liquid‐like structure of the systems are linked to the properties of PEG‐*g*‐PVAc. Nevertheless, the polymer structure and its constituting blocks can be tailored to adapt to different encapsulation conditions. Moreover, since amphiphilic polymers have been used to encapsulate hydrophobic compounds in different technological fields, and considering the biocompatibility of PEG and PVAc, the approach described in the present study can be easily implemented in a broader area of research, with special attention to the cosmetic, food, and pharmaceutical fields.

## Conflict of interest

The authors declare no conflict of interest.

## Supporting information

As a service to our authors and readers, this journal provides supporting information supplied by the authors. Such materials are peer reviewed and may be re‐organized for online delivery, but are not copy‐edited or typeset. Technical support issues arising from supporting information (other than missing files) should be addressed to the authors.

Supporting Information

Supporting Information
