# Supplementary material for: Rational Design of Sustainable Liquid Microcapsules for Spontaneous Fragrance Encapsulation
Source: Angew Chem Int Ed Engl. 2021 Sep 17;60(44):23849–57. doi: 10.1002/anie.202110446 (PMC8596835; doi:10.1002/anie.202110446)
Supplement: Supplementary file 1 — Supporting Information [file ANIE-60-23849-s001.pdf]

Angewandte Chemie
Eine Zeitschrift der Gesellschaft Deutscher Chemiker

Supporting Information

Rational Design of Sustainable Liquid Microcapsules for Spontaneous Fragrance Encapsulation

*Marianna Mamusa, Rosangela Mastrangelo, Tom Glen, Sergio Murgia, Gerardo Palazzo, Johan Smets, and Piero Baglioni**

anie_202110446_sm_miscellaneous_information.pdf
anie_202110446_sm_Movie.mp4

Table of contents

1. Materials and methods
2. Raman spectroscopy
3. Confocal laser scanning microscopy (CLSM) imaging
4. Cryo FIB/SEM image analysis
5. Small-angle X-ray scattering, optical microscopy, and rheology
6. NMR self-diffusion investigation
7. References

1. Materials and methods

Materials

Poly(ethylene glycol)-*graft*-poly(vinyl acetate) (PEG-*g*-PVAc) was a BASF product; the polymer is characterized by a PEG/PVAc weight ratio of 40/60, molecular weight M_n = 13.1 kDa (PDI 2.1), degree of branching of 1–2%.[1] For fluorescence correlation spectroscopy (FCS) experiments, PEG-*g*-PVAc was covalently labelled with rhodamine-B isothiocyanate (RBITC; mixed isomers, Sigma-Aldrich, Milan, Italy) according to a previously described procedure.[2] Alexa Fluor 568 for FCS calibration was purchased from ThermoFisher Scientific. All solvents, including fragrance compounds, were purchased from Sigma-Aldrich (Milan, Italy) and used as received. Water used in this work was Milli-Q grade (18.2 MΩ cm at 25 °C); D_2O (deuterium content > 99%) was used for NMR measurements.

Hansen Solubility Parameters (HSP)

Approximately 500 mg of PEG-*g*-PVAc was weighed in glass vials, and solvent was poured to obtain 10 %wt solutions. The vials were left in an orbital shaker at 25 °C for 24 h. A solvent was deemed "good" (score = 1) if capable of dissolving the polymer completely and "bad" (score = 0) if the polymer remained visibly undissolved. An intermediate score (= 2) was assigned to solvents that yielded cloudy solutions, such as butanol and ethanol. The list of solvents used, along with their HSP, is given in Table S1. All data

are part of the database provided with the software used in this work, HSPiP (Hansen Solubility Parameters in Practice, © 2008-20 Steven Abbott and Hiroshi Yamamoto, www.hansen-solubility.com).

Table S1. Solvents used in this work and their HSP (units are $MPa^{1/2}$). See main text for definitions.

Solvent	δD	δP	δH	Score	RED
Acetone	15.5	10.4	7	1	0.685
Acetonitrile	15.3	18	6.1	1	0.998
1-Butanol	16	5.7	15.8	2	1.147
n-Butyl Acetate	15.8	3.7	6.3	0	1.118
Chloroform	17.8	3.1	5.7	1	0.491
Eucalyptol	16.7	4.6	3.4	0	1.001
Cyclohexane	16.8	0	0.2	2	1.258
Diethyl Ether	14.5	2.9	4.6	2	1.269
Diethylene Glycol	16.6	12	19	2	1.719
Dimethyl Carbonate	15.5	8.6	9.7	1	0.729
Dimethyl Formamide	17.4	13.7	11.3	1	0.657
Dimethyl Sulfoxide	18.4	16.4	10.2	1	0.624
1,4-Dioxane	17.5	1.8	9	1	0.94
Dipropylene Glycol	16.5	10.6	17.7	0	1.483
Ethanol	15.8	8.8	19.4	2	1.014
Ethyl Acetate	15.8	5.3	7.2	1	0.854
Geraniol	16.9	4.2	7.6	1	0.794
Hexane	14.9	0	0	0	1.598
Isoeugenol	18.9	5.7	9.8	1	0.54
L-Carvone	17.5	5.8	3.7	1	0.646
d-Limonene	17.2	1.8	4.3	0	1.265
Linalool	16.8	2.9	6.9	1	0.913
Methyl Salicylate	18.1	8	13.9	1	0.298
2-Phenyl Ethanol	18.3	5.6	11.2	1	0.701

α-Pinene	16.9	1.8	3.1	0	1.289
Propylene Carbonate	20	18	4.1	1	0.835
Terpinyl Acetate	16.8	2.8	3.5	0	1.333
Toluene	18	1.4	2	1	0.998
Undecylenic Aldehyde	16.1	5.0	3.3	0	1.153

The solubility parameter "distance" *Ra* between two materials, based on their respective partial solubility parameter components, is given by:[3]

$$(Ra)^2 = 4(\delta D_2 - \delta D_1)^2 + (\delta P_2 - \delta P_1)^2 + (\delta H_2 - \delta H_1)^2 \quad \text{(S1)}$$

This equation is used to calculate the best sphere encompassing the good solvents and leaving out the bad ones (see main text). For each solvent, the RED (= *Ra* / R_0, where R_0 is the interaction radius) number is also given in Table S1 as a measure of the fit quality: good solvents have RED < 1 (they are inside the sphere), while bad solvents have RED > 1 (they are outside the sphere).

Phase diagrams

PEG-*g*-PVAc/perfume/water ternary phase diagrams were constructed by weighing the appropriate amounts of water, polymer, and perfume in a glass vial using an analytical balance (Radwag AS R2, accuracy ± 0.1 mg); the polymer was first molten at 50 °C for ease of manipulation. Samples were vortexed until homogenization and stabilized at 25 °C in an oven for 14 d.

Sample shown in movie for Supporting Information

A movie is provided as Supporting Information to demonstrate the spontaneous formation of the microcapsules. The clip shows a sample that was prepared as follows: a 4-mL clean glass vial was placed in a holding support to immobilize it; 90 mg of molten PEG-*g*-PVAc was added to the glass vial, and the polymer was allowed 1 h to cool completely to 25 °C. At this point, 100 mg of 2-phenyl ethanol (PE) was injected in the vial, followed by 810 mg Milli-Q water. The vial was immediately capped to avoid evaporation of the contents. The evolution of the sample was filmed with a Canon EOS 60D camera equipped with a 100-mm f/2.8 Macro lens.

Confocal scanning laser microscopy (CLSM)

CLSM imaging was carried out using a Leica TCS SP8 confocal microscope (Leica Microsystems GmbH, Wetzlar, Germany). Samples were placed in appropriate wells (Lab-Tek Chambered 1.0 Borosilicate Coverglass System, Nalge Nunc International, Rochester, NY USA). A 63× water immersion objective was used to image all samples. Rhodamine-B was excited at 561 nm with a DPSS laser, and the fluorescence emission was acquired using a Hybrid SMD detector in the 571–600 nm range.

Fluorescence Correlation Spectroscopy (FCS)

FCS measurements of the RBITC-labeled polymer were performed on a Leica TCS SP8 confocal microscope (Leica Microsystems GmbH, Wetzlar, Germany) equipped with a 63× water immersion objective. RBITC (50 nM in the polymer aqueous solution) was excited with a DPSS 561 laser line (561 nm), while the fluorescence signal was collected with a Hybrid SMD detector (571–630 nm). The diffusion of the dye Alexa Fluor 568 in water was used in the calibration procedure to determine the confocal volume.[4]

FCS curves were averages of 15 acquisitions. Fitting was performed considering a three-dimensional Brownian diffusion of the labeled species across a 3D-ellipsoidal Gaussian volume:

$$G(\tau) = \frac{1}{N}\left[\left(1+\frac{\tau}{\tau_D}\right)^{-1}\left(1+\frac{\tau}{S^2\tau_D}\right)^{-1/2}\right] \qquad \text{(S2)}$$

where *N* is the average number of fluorescent molecules detected inside the confocal volume ($N = CV$, with $V = \pi^{3/2}{w_0}^3 S$ and C the concentration), τ_D is the decay time, and $S = z_0/w_0$ is the ratio between the axial and the lateral dimensions of the confocal volume, determined through the calibration procedure with Alexa 568. The diffusion coefficient *D* of the fluorescent molecules can be obtained from the relationship:

$$\tau_D = \frac{{w_0}^2}{4\mathrm{D}} \qquad \text{(S3)}$$

Confocal Raman microscopy

Raman analysis and mapping were performed on a Renishaw InvIa Qontor confocal MicroRaman system equipped with 785 nm (solid state type, IPS R-type NIR785, 100 mW, 1200 l/mm grating) and 532 nm (Nd:YAG solid state type, 50 mW, 1800 l/mm grating) lasers, front-illuminated CCD camera (256 × 1024 px, working temperature −70 °C) and a research-grade Leica DM 2700 microscope.

References for pure compounds were collected using the 785 nm excitation wavelength for PEG-*g*-PVAc and 532 nm excitation wavelength for the fragrances; Raman spectra were recorded in the wavenumber range from 100 to 3500 cm^{-1} using the extended range mode. Bidimensional maps were acquired using a long working distance 50× objective in high-confocality and static spectral range modes. Spectra were acquired with steps of 0.5 to 1 μm (depending on the sample) along the x-y plane. Typical acquisition times per point were 1 or 2 s, acquiring a single scan. Raw data were processed using Renishaw software WiRE v.5.2 for maps generation.

Cryogenic Focused Ion Beam (FIB) Scanning Electron Microscopy (SEM) and image analysis

Cryo FIB/SEM analysis was carried out using a Zeiss Crossbeam 550. Samples were placed in 3-mm carriers and frozen using a Leica HPM100 high-pressure freezer. These samples were then mounted under liquid nitrogen and transferred into a Quorum Technologies PP3010 cryo preparation chamber under vacuum. A platinum coating ~25 nm thick was deposited using the sputter coater built into the cryo preparation chamber. Cross-sections were prepared by milling a rough trench with the beam at 30 kV and 7 nA before polishing with a reduced beam current of 700 pA. SEM images were acquired using an accelerating voltage of 2 kV and a beam current of 75 pA with the in-lens detector. Cross-sectional images were acquired using the SEM at an angle of 54°. Images were made clearer through Fast Fourier Transform filtering to remove some curtaining and charging, followed by normalizing local contrast across 40 pixels with a standard deviation of 3. This image processing was done using FIJI (ImageJ).[5] The same software was used to measure interlamellar spacing in the FIB/SEM image shown in the main text (Fig. 6F). Namely, 8 lines were drawn on the figure along radial directions of the onion-like objects. Then, the linear profile of light intensity along each line was calculated: the distances between neighboring minima, calculated with MATLAB, describe the thickness of the lamellae. Approximately 200 characteristic distances were obtained, which spontaneously clustered in 15 values.

Small-angle X-ray Scattering (SAXS)

SAXS measurements were performed on a Xeuss 3.0 HR (Xenocs) instrument, featuring a GeniX 3D Cu High Flux Very Long Focus (HFVL) Complete x-ray generator equipped with a high brightness X-ray tube (30W/40μm) and a FOX 3D single reflection multilayer optic. The signal was collected with a Dectris Eiger 2R 1M hybrid photon counting detector (pixel dimension of 75 × 75 μm^2). 2D SAXS images were collected, and circularly averaged through the XSACT software, to be expressed as Intensity vs. Q (where the modulus of the scattering vector is defined as $Q = (4\pi/\lambda)\sin\theta$, with 2θ the scattering angle). SAXS data were collected at two sample-to-detector distances: 450 mm and 1800 mm. The 2 data sets were corrected by subtracting the scattering intensities of the continuous medium/empty cell (normalizing for the time of measurement and the relative transmission factors) and the combined in a single curve trough the XSACT software, to cover a final Q-range between 0.004 Å^{-1} and 0.6 Å^{-1}. Glassy carbon was used to convert the intensity in absolute scale by considering the thickness of each sample[6]. Analyzed samples were freshly prepared and kept at 25°C for 10 days at least. For the measurements, gel-like samples were placed in a steel "gel-type" sample holder, using Kapton tape as windows, while milky liquids were poured in glass capillaries (1.5 mm diameter). Measurements were performed at 25°C using the "in air" mode to minimize evaporation.

The SAXS curve of a representative sample, containing capsules (fig. 6 E,F, main text), was fitted according to a core-shell form factor (SASView Software v.4.2.2, http://www.sasview.org/):

$$P(Q) = \frac{Scale}{V_S} F^2(Q) + bkg \tag{S4}$$

$$F(Q) = \frac{3}{V_S}\left[V_C(\rho_C - \rho_S)\,\frac{sin(QR_C)-QR_C\cos(QR_C)}{(QR_C)^3} + V_S(\rho_S - \rho_{Solv})\,\frac{sin(QR_S)-QR_S\cos(QR_S)}{(QR_S)^3}\right] \tag{S5}$$

Where the subscripts are: C (core), S (Shell), Solv (Solvent). ρ are the Scattering Length Densities (SLD). V_S is the total volume of the particle, and $R_S = Rc + t$ is the total radius of the particle, with t the shell thickness.

A Shultz distribution of radii, to account for particles polydispersity, was used:

$$f(R) = (z+1)^{z+1}x^z\,\frac{\exp[-(z+1)x]}{R_{mean}\Gamma(z+1)} \tag{S6}$$

with z = 1 / (PDI^2 - 1), i.e. a function of polydispersity, PDI = σ/R_{avg}, with σ^2 the variance of the distribution, R_{mean} the mean radius, x = R/R_{mean} and Γ the Gamma function.

The interaction between polymer particles in the multicompartment capsules was interpreted according to a hard-sphere structure factor[7]:

$$S(Q) = [1 + \frac{24\phi_{HS}X(Q)}{R_{HS}}]^{-1} \quad \text{(S7)}$$

being $X(Q)$ a function of the following type:

$$X(Q) = \alpha K_0(2QR_{HS}) + \beta K_1(2QR_{HS}) + \frac{\phi_{HS}\alpha}{2} K_3(2QR_{HS}) \quad \text{(S8)}$$

$$K_0(2QR_{HS}) = j_1(2QR_{HS}), \qquad K_1(2QR_{HS}) = \frac{2(2QR_{HS})\sin(2QR_{HS}) + (2-(2QR_{HS})^2)\cos((2QR_{HS})-2)}{(2QR_{HS})^3}$$

$$K_3(2QR_{HS}) = \frac{-(2QR_{HS})^4\cos(2QR_{HS}) + 4\{[3(2QR_{HS})^2-6]\cos(2QR_{HS}) + [(2QR_{HS})^3 - 6(2QR_{HS})\sin(2QR_{HS}) + 6]\}}{(2QR_{HS})^5}$$

$$\alpha = \frac{(1+2\phi_{HS})^2}{(1-\phi_{HS})^4}, \qquad \beta = -6\phi_{HS}\frac{(1+\phi_{HS}/2)^2}{(1-\phi_{HS})^4}$$

R_{HS} the hard-sphere radius and ϕ_{HS} the hard spheres volume fraction. The potential can be described as follows:

$$U(R) = \begin{cases} \infty \; if \; r < 2R \\ 0 \; if \; r \geq 2R \end{cases}$$

In the fitting procedure, the SLD of the linalool and water were fixed.

Table S2. Results of SAXS data modelling with a core-shell form factor and a hard-sphere structure factor.

Parameter	Value	Error
Scale	0.0662	0.0001
Background	0.0046	0.0001
Core Radius, R_c (nm)	19.1	0.1
PDI	0.170	0.001
Shell thickness, t (nm)	6.8	0.1
SLD_{Core} (10^{-6} Å^{-2})	8.0	----

SLD_{Shell} (10^{-6} Å^{-2})	9.8	0.1
$SLD_{Solvent}$ (10^{-6} Å^{-2})	9.4	----
Volume Fraction	0.313	0.001

Rheology

The rheological behavior of characteristic gel-like, birefringent samples was investigated with a Discovery HR-3 rheometer from TA Instruments (steel parallel plate-plate geometry, 20 mm), equipped with a Peltier temperature control system. All samples were equilibrated at 25 °C before the measurements, and they were tested at the same temperature. The linear viscoelastic regime was identified through amplitude sweeps collected between $9 \cdot 10^{-3}$–10% strain at 0.5 rad/s. Frequency sweeps were obtained in the range of frequencies ω = 0.1–300 rad/s. From the same experiment, the profiles of the complex viscosity, η*, were obtained for each sample. The values of the storage and loss moduli (G' and G'', respectively) and η* shown in the plots are averages of four repeated measurements.

NMR self-diffusion

^{1}H NMR measurements were performed in deuterated water at 25 °C using a Bruker Avance 300 MHz spectrometer at the operating frequency of 300.13 MHz while controlling the temperature (± 0.5 °C) with a BVT 3000 unit. For the determination of self-diffusion coefficients *D*, a Bruker DIFF30 probe supplied by a Bruker Great 1/40 amplifier was used. *D* coefficients were obtained using the pulse-gradient stimulated echo (PGSTE) sequence by varying the gradient strength (*g*) while keeping the gradient pulse length (*δ*) and the gradient pulse intervals constant within each experimental run. The data were fitted according to the Stejskal-Tanner equation:

$$\frac{I}{I_0} = exp(-Dq^2t) \qquad \text{(S9)}$$

where I and I_0 are the signal intensities respectively in the presence and absence of the applied field gradient, $q = \gamma g\delta$ is the so-called scattering vector (γ being the gyromagnetic ratio of the observed nucleus), $t = (\Delta - \delta/3)$ is the diffusion time, and Δ is the delay time between the encoding and decoding gradients. Errors were estimated lower than 2% on the basis of repeated measurements.

2. Raman spectroscopy

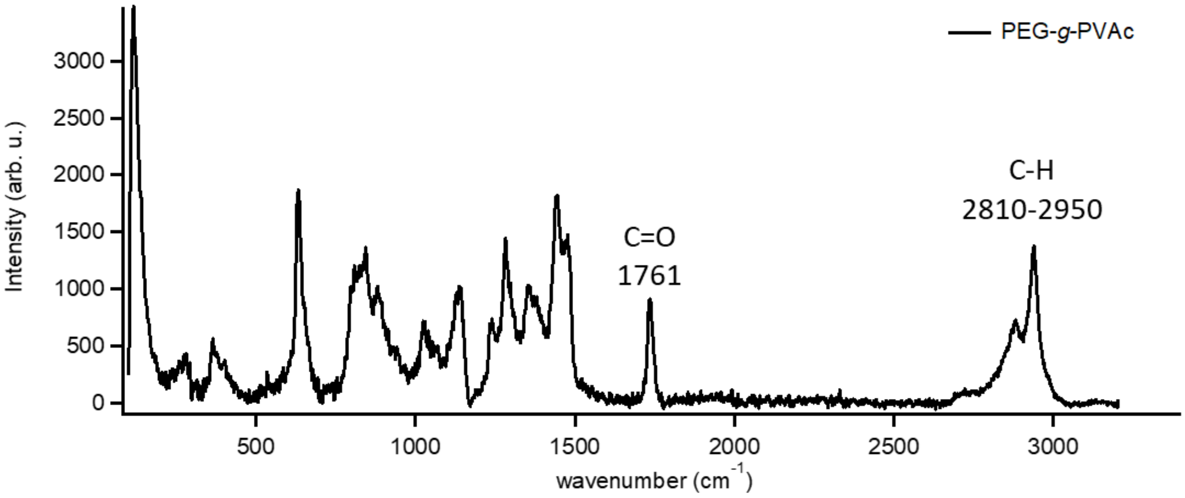

Figure S1. Raman spectrum of pure PEG-*g*-PVAc.

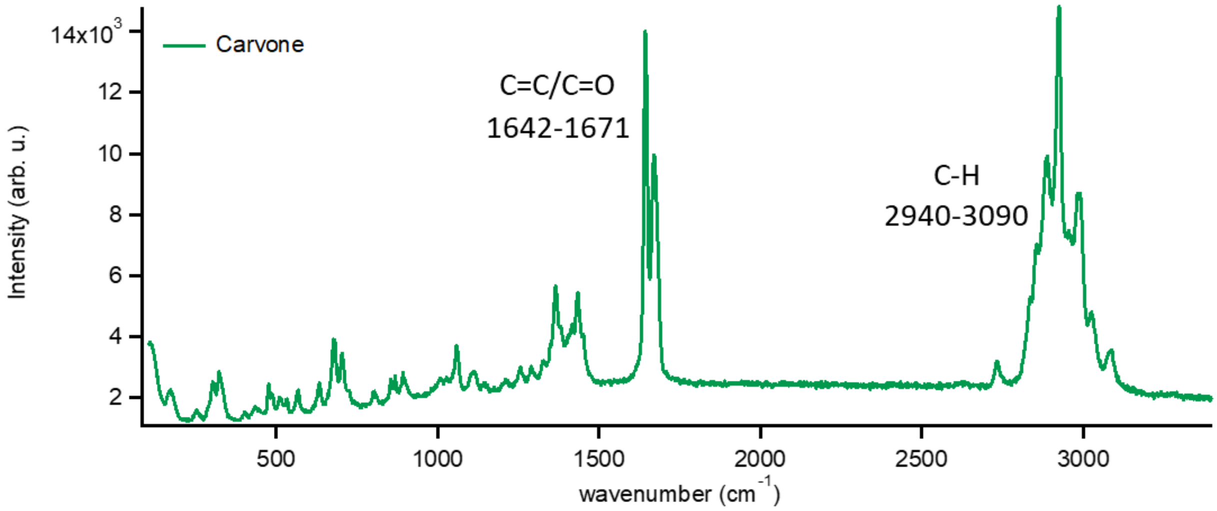

Figure S2. Raman spectrum of pure L-carvone.

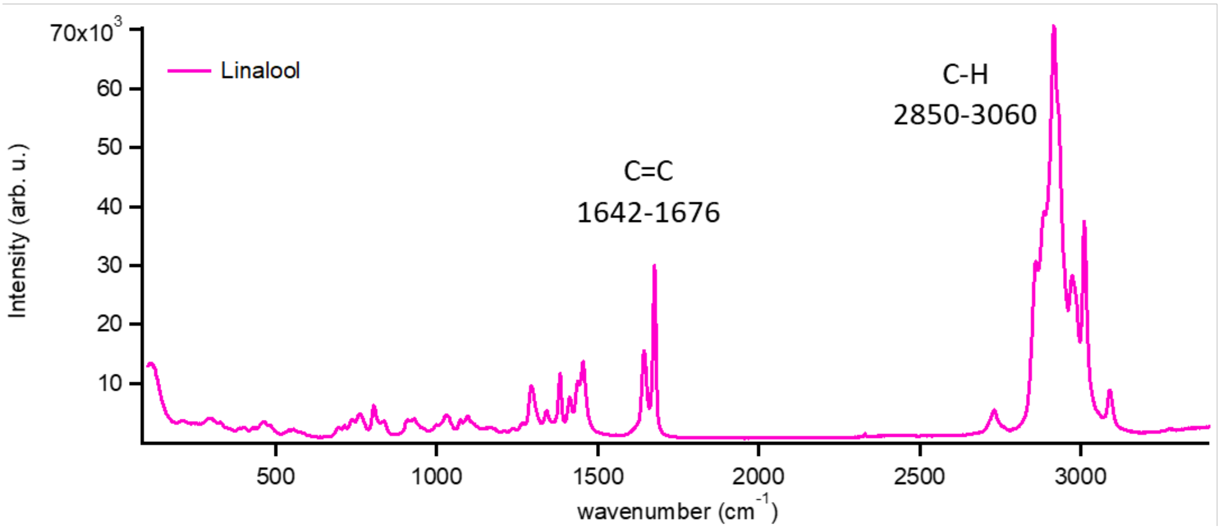

Figure S3. Raman spectrum of pure linalool.

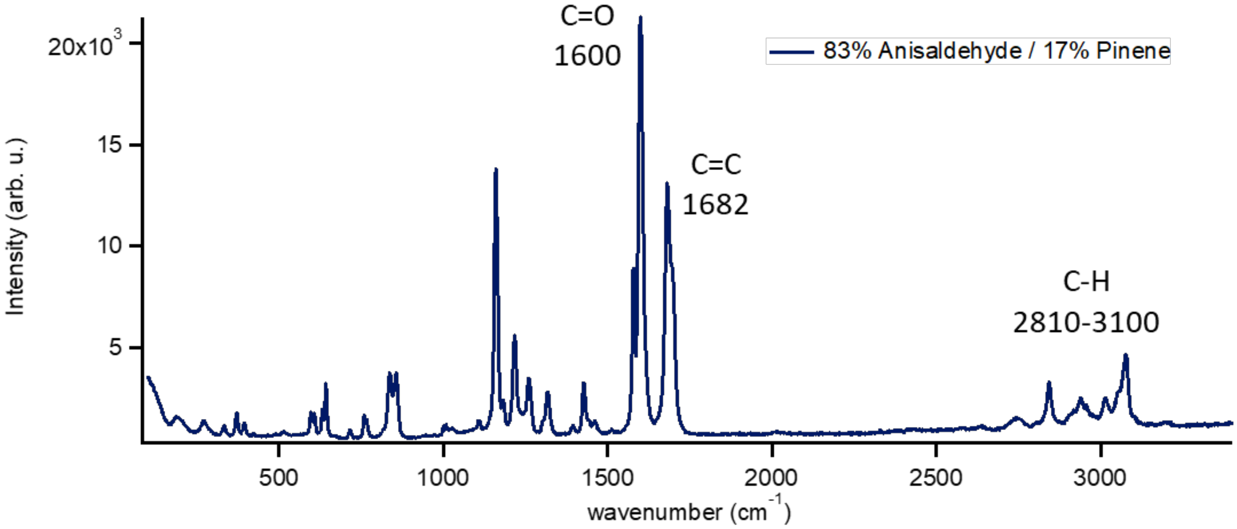

Figure S4. Raman spectrum of the mixture 83% anisaldehyde / 17% α-pinene.

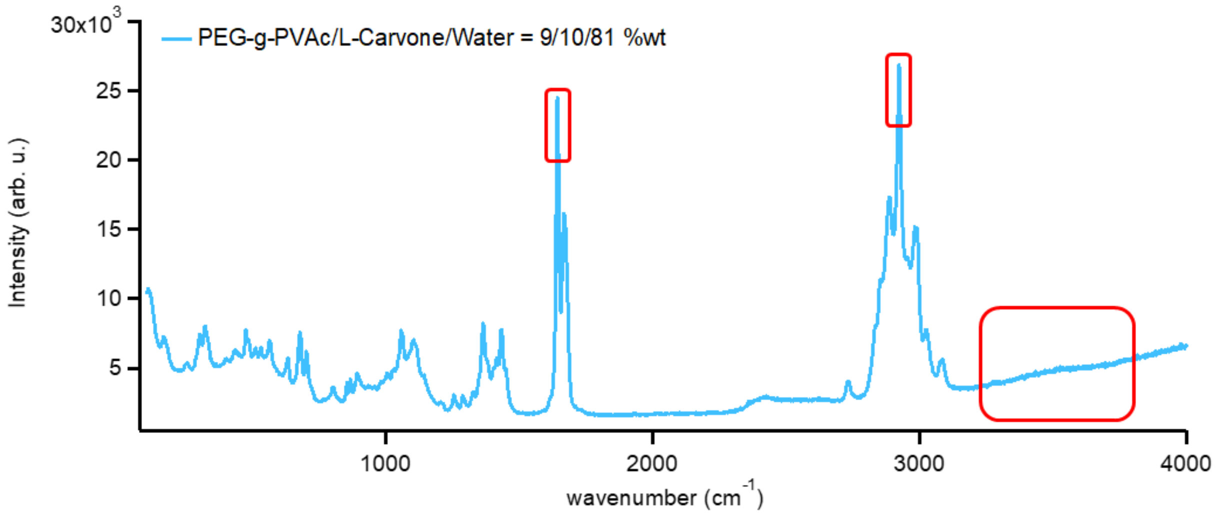

Figure S5. Representative Raman spectrum taken inside a capsule in the PEG-*g*-PVAc/L-carvone/water system. Red squares indicate peaks used in 2D mapping.

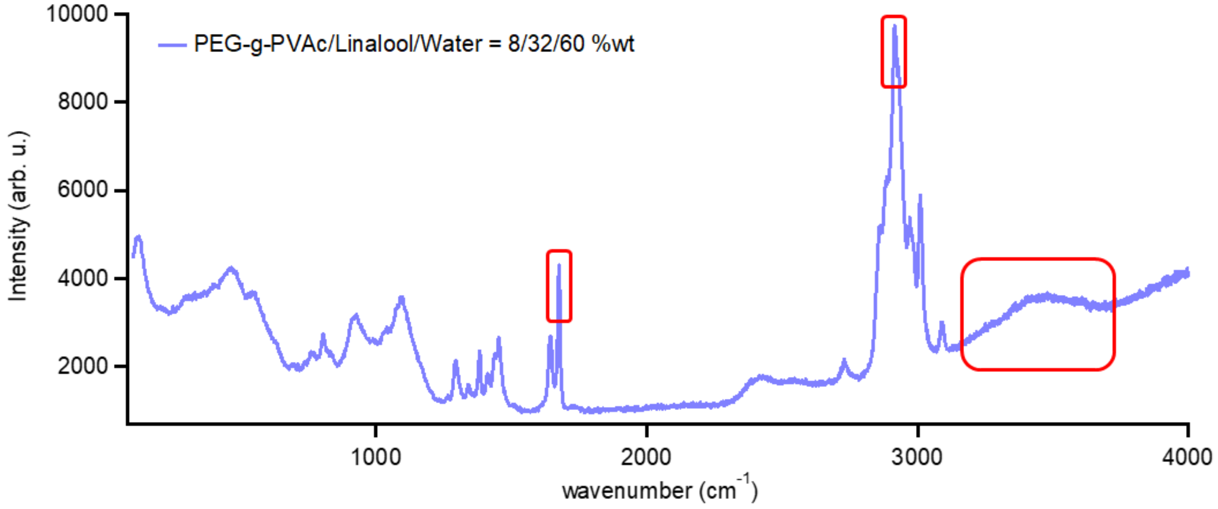

Figure S6. Representative Raman spectrum taken inside a capsule in the PEG-*g*-PVAc/linalool/water system. Red squares indicate peaks used in 2D mapping.

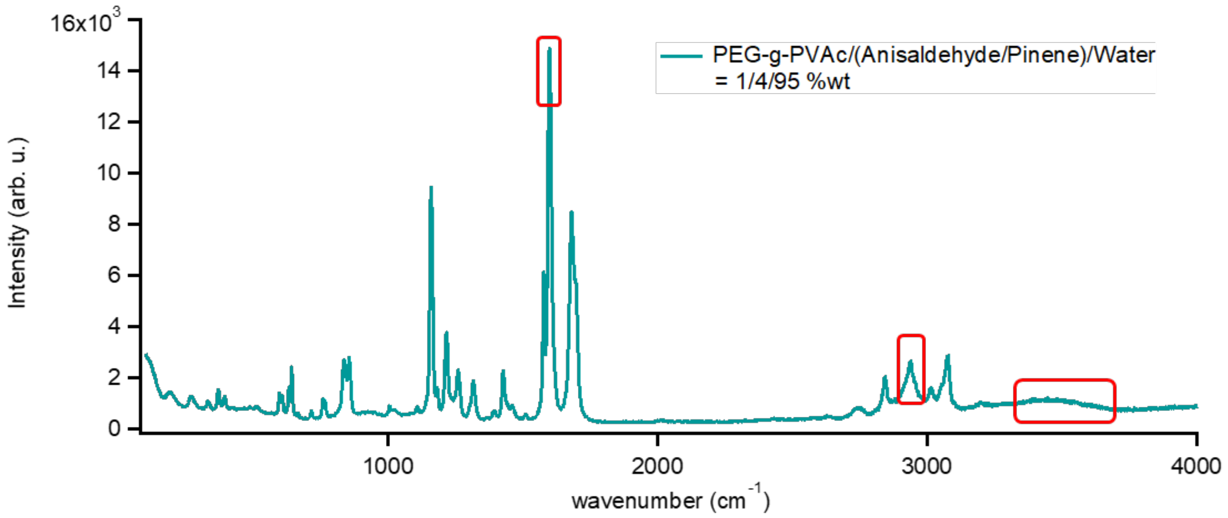

Figure S7. Representative Raman spectrum taken inside a capsule in the PEG-*g*-PVAc/anisaldehyde/α-pinene/water system. Red squares indicate peaks used in 2D mapping.

3. Confocal laser scanning microscopy (CLSM) imaging

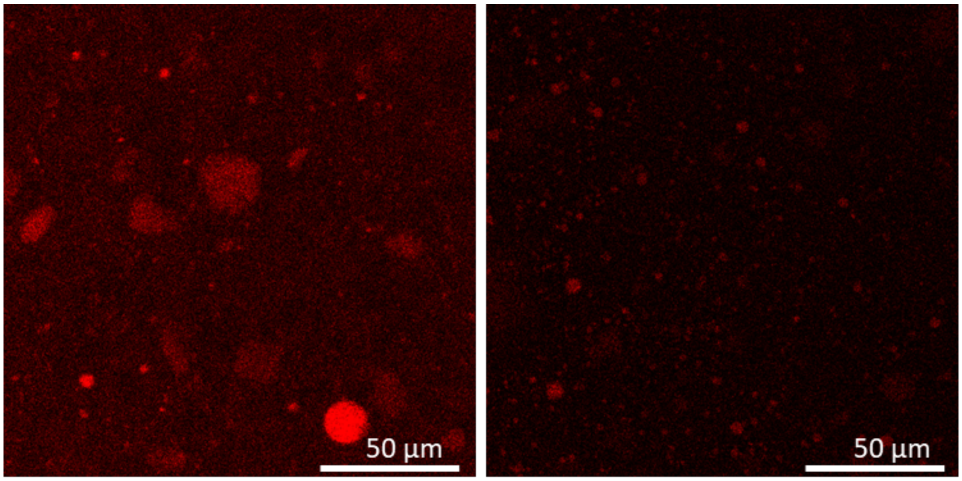

Figure S8. CLSM images of a representative sample taken in the droplet region of the PEG-*g*-PVAc/isoeugenol/water system. The red signal originates from the RBITC-labelled polymer, excited with a 561 nm laser line.

4. Cryo FIB/SEM image analysis

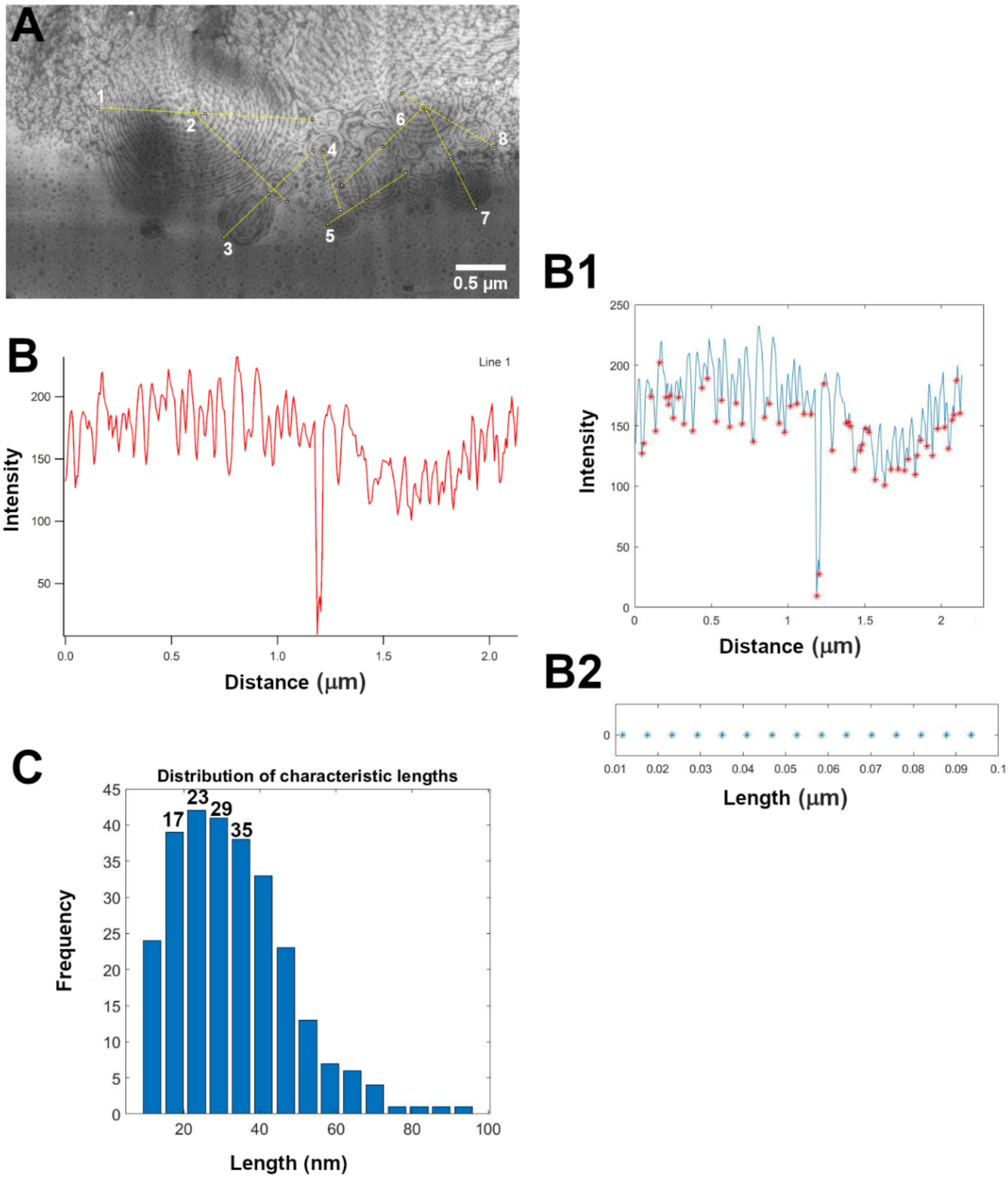

Figure S9. Quantitative image analysis on onion-like phases observed in a PEG-g-PVAc/linalool/water system, performed with the software Image J. Interlamellar spaces were quantified by drawing radial lines on the observed objects (A); along each line, the intensity of the greyscale was measured (B shows the profile obtained for line 1). Minima were found on each profile using MATLAB (B1), and interlamellar spaces were calculated by subtracting the values of neighboring minima. The set of obtained lengths spontaneously clustered in 15 repeated values (B2). The frequency of each value is shown in panel C. The most recurrent values are in agreement with the interlamellar spacings, d, calculated from SAXS experiments on structured samples, in the present (d = 22–28 nm, see Fig. S12) and in previous work.

5. Small-angle X-ray scattering (SAXS), optical microscopy, and rheology

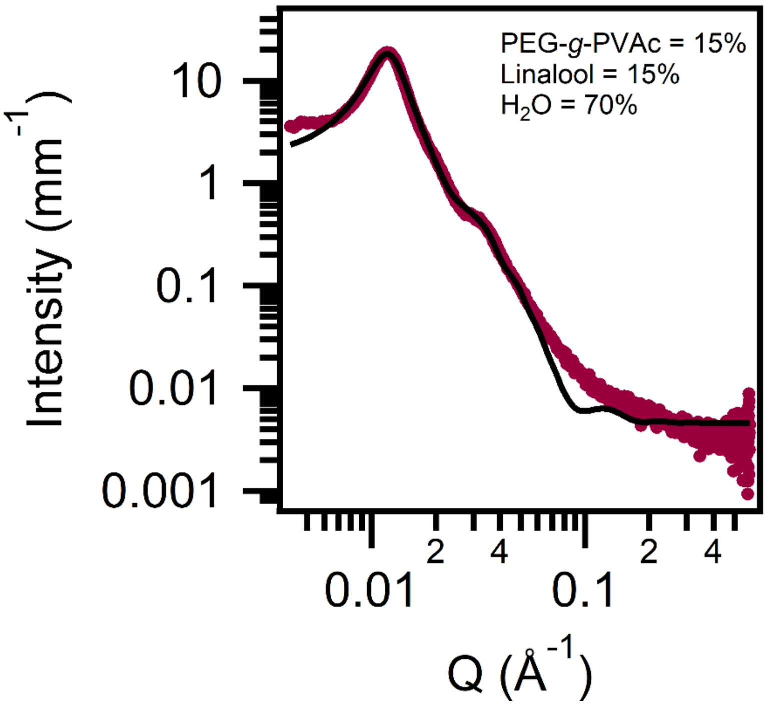

Figure S10. SAXS curve (red markers) and fitting (black line) for a sample taken in the droplet region of the PEG-*g*-PVAc/Linalool/water system (concentrations indicated in the figure legend are %wt). The fitting accounts for the presence of core-shell particles, *i.e.*, those constituting the multicompartment capsules.

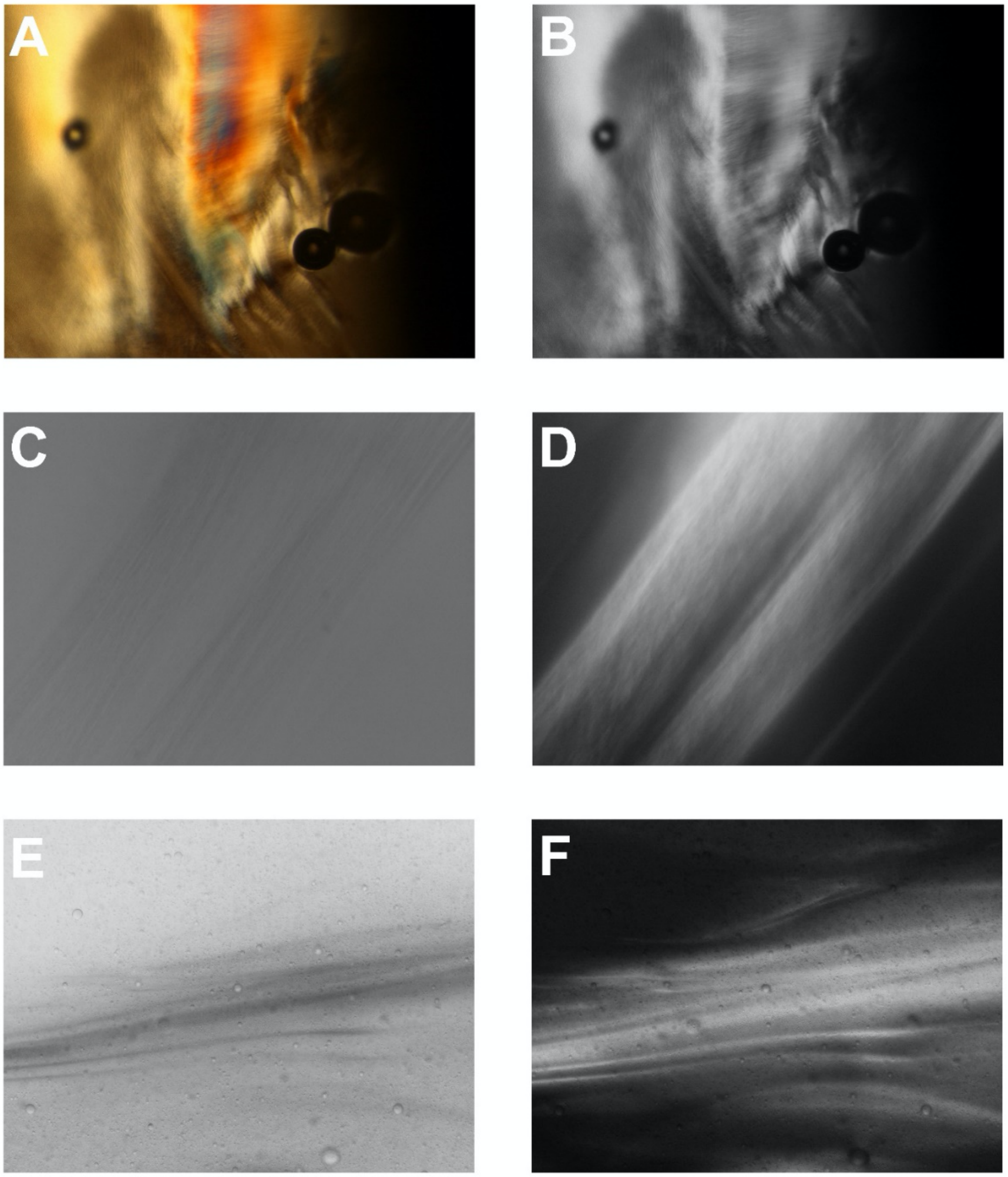

Figure S11. Optical micrographs in normal (left) and polarized (right) light, representative of areas of weak local order in the analyzed samples. A-B) Sample containing 31.5 %wt PEG-*g*-PVAc, 13.5 %wt isoeugenol, and 55 %wt water; C-D) sample containing 27 %wt PEG-*g*-PVAc, 18 %wt linalool, and 55 %wt water; E-F) sample containing 24 %wt PEG-*g*-PVAc, 16 %wt anisaldehyde/pinene, and 60 %wt water. Magnification: 10×.

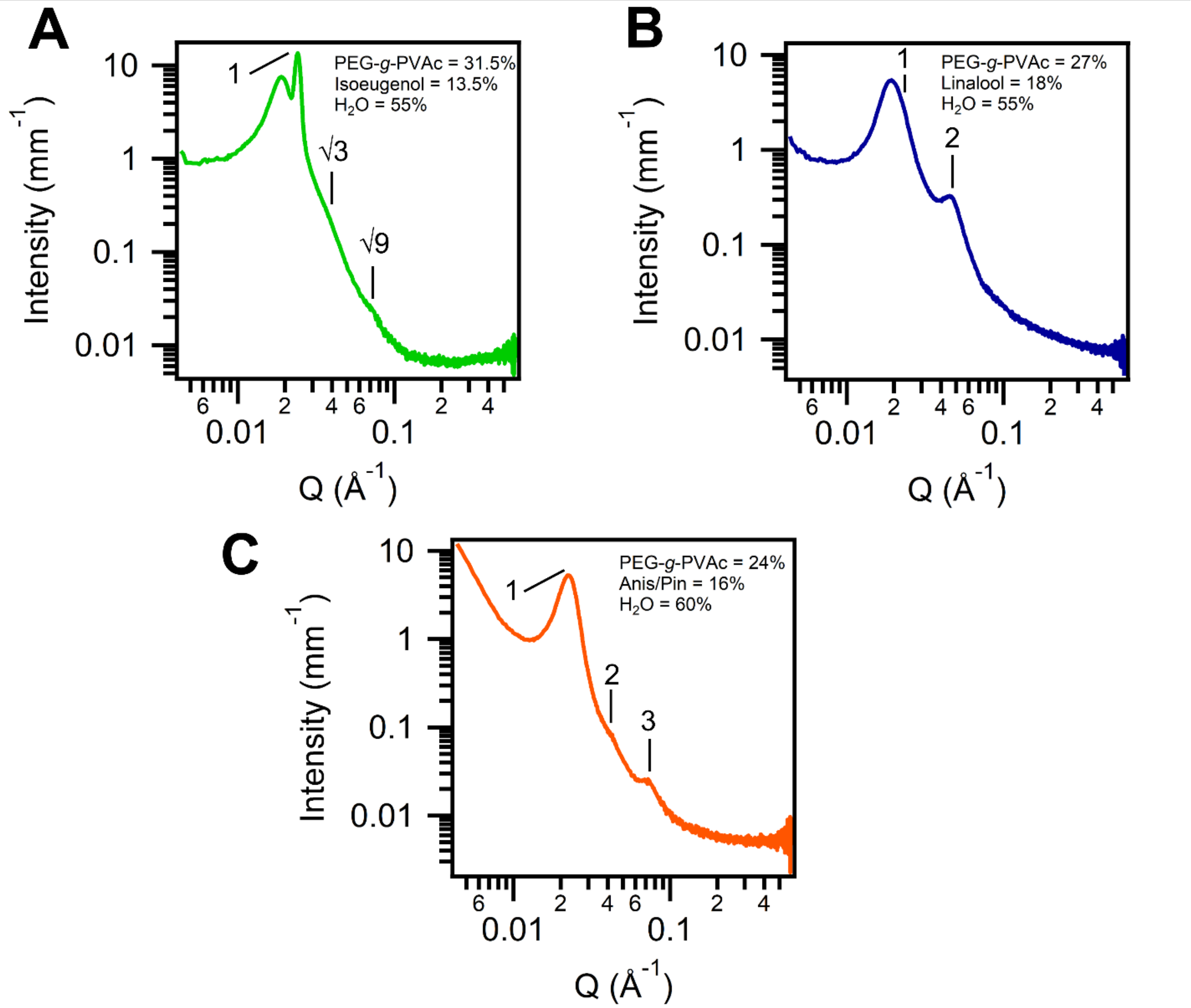

Figure S12. SAXS curves obtained for samples containing A) isoeugenol, B) linalool, and C) anisaldehyde/pinene. Concentration in the figure legends are %wt. The samples are the same whose micrographs are shown in Fig. S11, and they were chosen in regions of the phase diagrams adjacent to those of capsules ("B" regions in Figs. 2 and 7, main text). The correlation peaks were identified by comparing SAXS and rheology data (Fig. S14; see the main text for details).

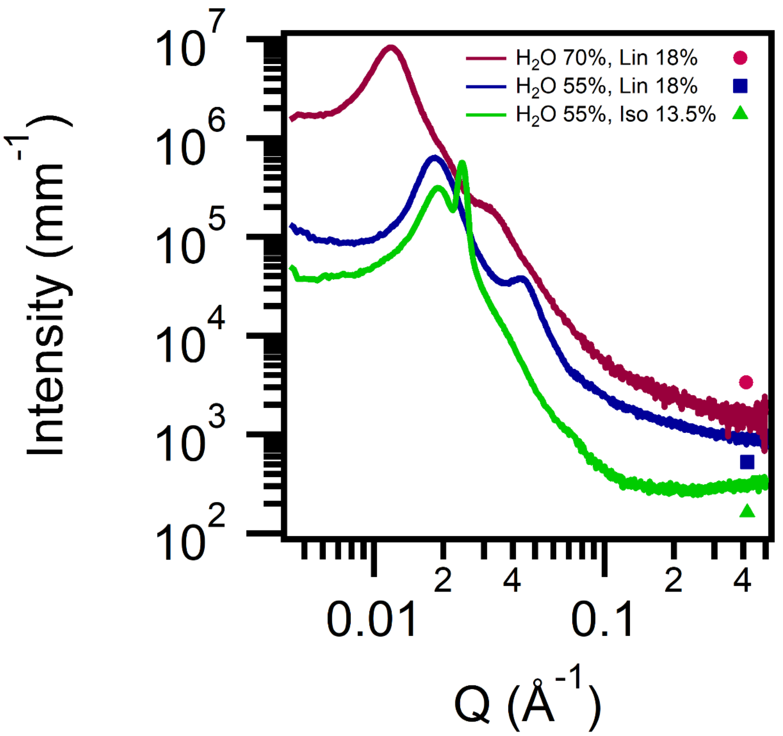

Figure S13. Comparison of SAXS curves shown in Figs. S10 and S12; concentrations in the figure legend are %wt. The sample containing capsules (70% H_2O and 18% linalool, indicated by a red circle) exhibits a main peak that can be related to the first broad peak observed in the two more structured samples. The samples containing linalool (55% H_2O 18% linalool, blue square) and isoeugenol (55% H_2O and 13.5% isoeugenol, green triangle) show a similar broad peak at low Q. This suggests that structured samples contain at least another phase, probably consisting of polymer capsules.

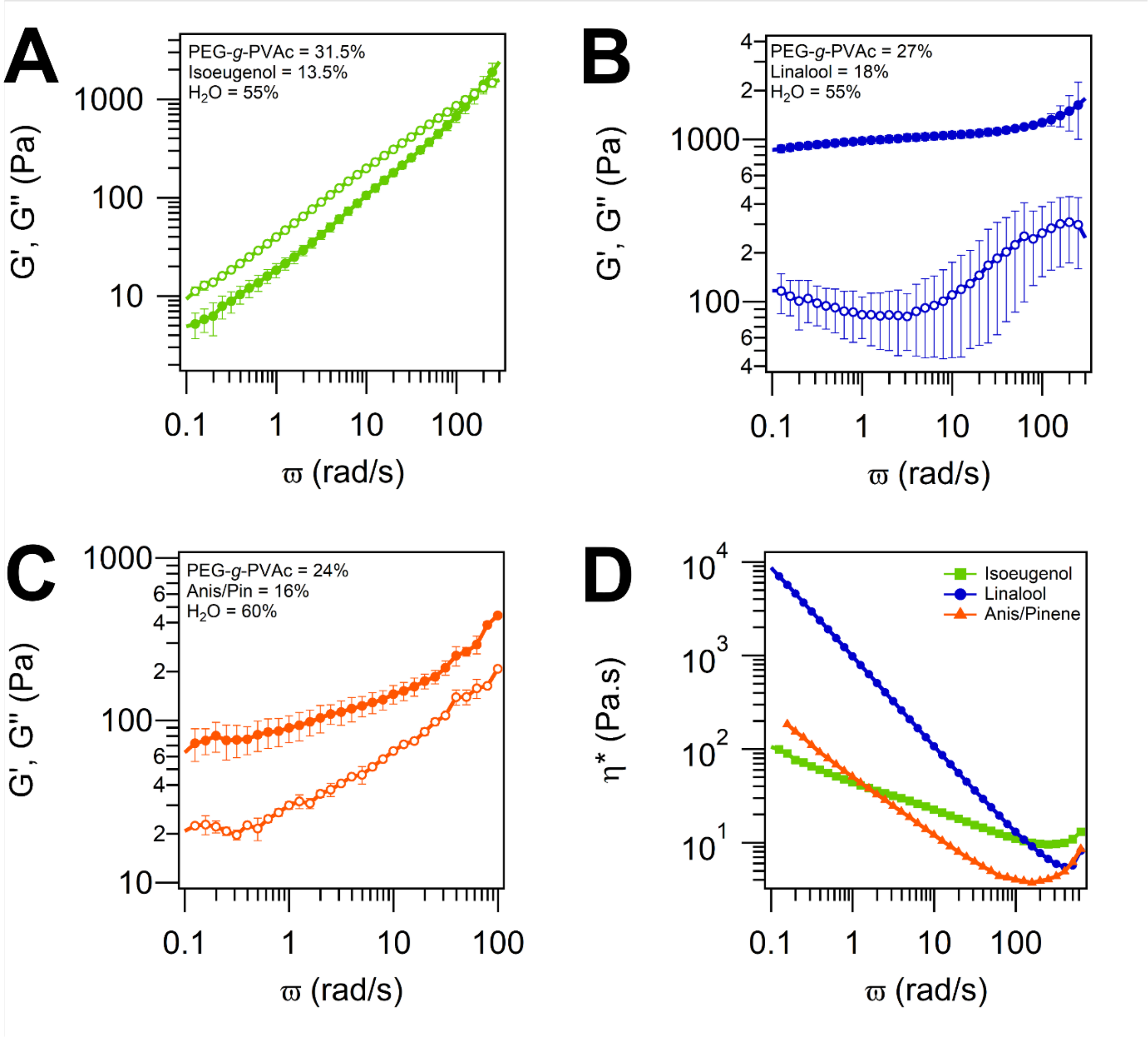

Figure S14. Frequency sweeps, obtained through rheology measurements, of the samples shown in Figs. S11 and S12: A) Isoeugenol; B) Linalool; C) Anisaldehyde/Pinene. The storage modulus, G', is indicated with filled markers, while the loss modulus, G'', is indicated by empty markers. The sample containing isoeugenol (A) behaves as a viscous fluid and can be identified as a mesophase characterized by a hexagonal order. Samples containing linalool (B) or anisaldehyde/pinene (C) display a gel-like behavior, with G' > G'' at all frequencies, *i.e.*, the fingerprint of lamellar phases. D) Profiles of complex viscosity for the three samples; all samples display shear-thinning behavior, even if it is more pronounced in the lamellar phases.

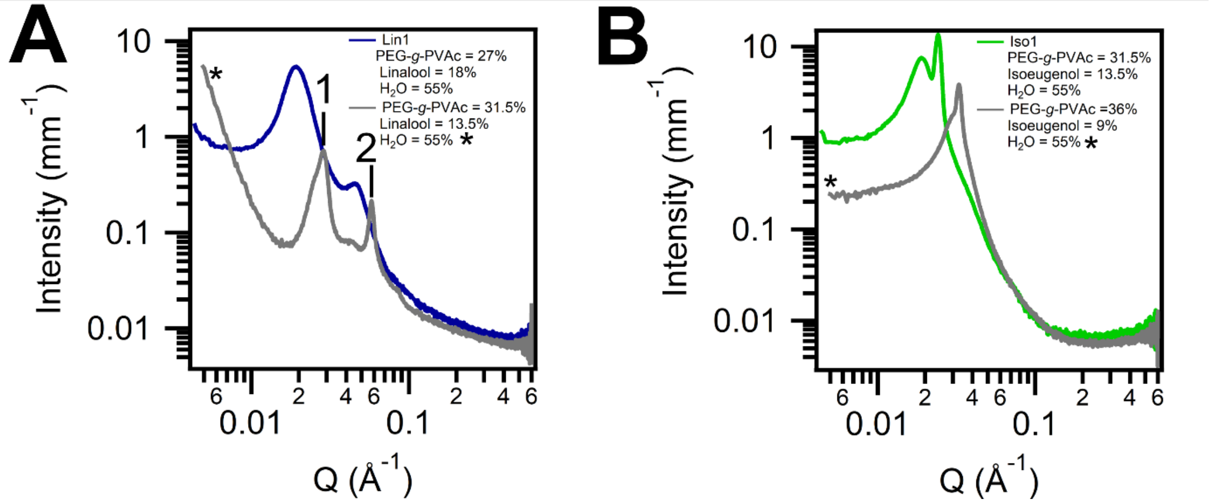

Figure S15. Effect of the increase in polymer:perfume ratio at constant water content (55 %wt) on the SAXS profiles of the samples containing A) linalool and B) isoeugenol (Lin1 and Iso1 are the curves shown in Fig. S12). More concentrated samples are indicated by (*) in the figure. Characteristic peaks shift to higher Q values, suggesting a shrinkage of the ordered structures. The concentrated linalool sample displays two sharp peaks, related to the prevalence of the lamellar phase in the sample.

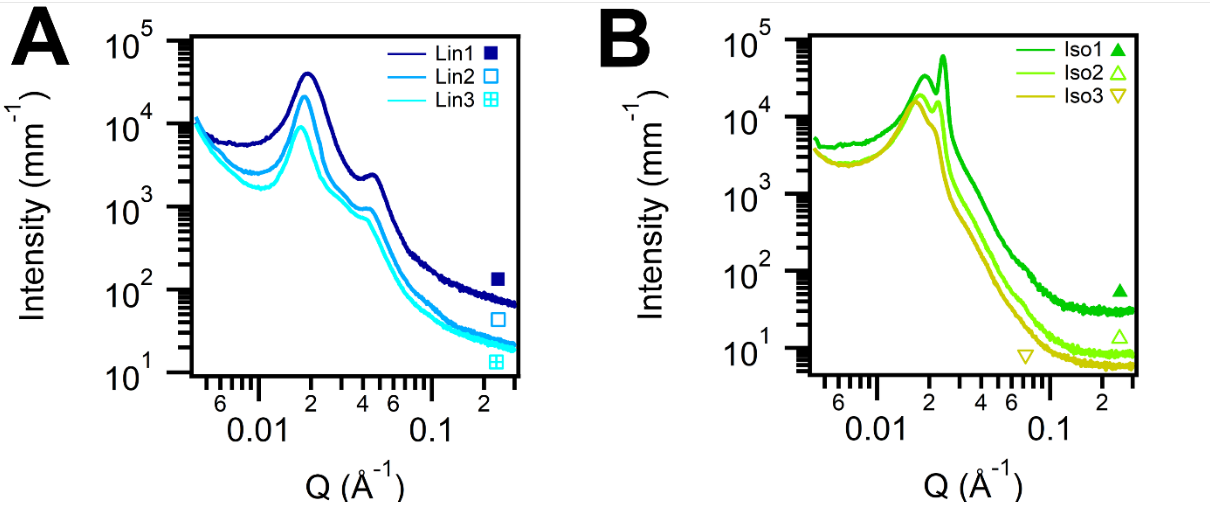

Figure S16. Effect of the increase in water content at a constant polymer:perfume ratio on the SAXS profiles of the samples containing A) linalool and B) isoeugenol (Lin1 and Iso1 are the curves shown in Fig. S12). Water increases from 55 %wt to 60 %wt and 65 %wt. The broad peak in the low-Q region is not significantly altered by dilution but shifts to lower Q values, suggesting the swelling of the capsules present in the samples. The structural peaks become less intense with increasing dilution, suggesting the partial disruption of ordered areas.

6. NMR self-diffusion investigation

Table S3. Self-diffusion coefficients (in m^2/s) of perfume, D_2O, and polymer i) in the coacervate and ii) in mixtures of perfume in water, as measured by NMR. PE = 2-phenyl ethanol; Car = L-carvone; Lin = linalool; HOD = residual proton of deuterated water.

Sample	Perfume (PE/Car/Lin)	HOD	Polymer (biexponential deconvolution)	
PE (aq) [0.008 M]	$6.7 \cdot 10^{-10}$	$1.9 \cdot 10^{-09}$	----	----
CAR (aq) [0.008 M]	$5.9 \cdot 10^{-10}$	$1.9 \cdot 10^{-09}$	----	----
LIN (aq) [0.008 M]	$4.4 \cdot 10^{-10}$	$1.9 \cdot 10^{-09}$	----	----
PEG-*g*-PVA/PE/D_2O 9/10/81 wt%	$1.2 \cdot 10^{-10}$	$1.5 \cdot 10^{-09}$	$4.5 \cdot 10^{-12}$	$1.9 \cdot 10^{-13}$
PEG-*g*-PVA/Car/D_2O 9/10/81 wt%	$2.1 \cdot 10^{-11}$	----	$2.8 \cdot 10^{-11}$	$3.4 \cdot 10^{-12}$
PEG-*g*-PVA/Lin/D_2O 10/15/75 wt%	$5.6 \cdot 10^{-11}$	$1.2 \cdot 10^{-09}$	$1.5 \cdot 10^{-11}$	$2.1 \cdot 10^{-12}$

The self-diffusion coefficients of the perfumes were analyzed assuming a two-sites model where the perfume molecules are in fast exchange between the aqueous bulk and a micrometer-sized droplet. In such a case, the observed self-diffusion coefficient is given by Lindman's law:

$$D_{obs} = P_b D_b + (1 - P_b) D_f \tag{S10}$$

where D_f and D_b are the diffusivities of the free and bound states, respectively, and *P* indicates the volume fraction of the component.

The D_f value was measured in a separate experiment in a D_2O solution of perfume (0.008 M). The diffusion of the perfume within the particle can be assumed to be $D_b \approx 0$ because of the large size of the particle, so that the term $P_b D_b$ may be neglected; this leaves $D_{obs} \approx (1 - P_f) D_f$. The concentration of free perfume molecules in the bulk can be evaluated as $[perfume]_f = P_f [perfume]_{tot}$, where $[perfume]_f$ and $[perfume]_{tot}$ are the concentrations (in g/L) of the perfume with respect to the volume of (heavy) water. Using the data of Table S3, the following results were obtained:

Table S4. Volume fraction and concentration (in g/L) of free perfume in three-component systems, obtained *via* Eq. S9. PE = 2-phenyl ethanol; Car = L-carvone; Lin = linalool.

Sample	Fraction of free perfume	Concentration of free perfume
PEG-*g*-PVA/PE/D_2O 9/10/81 wt%	0.18 ± 0.02	24 ± 2 g/L
PEG-*g*-PVA/Car/D_2O 9/10/81 wt%	0.035 ± 0.005	4.7 ± 0.7 g/L
PEG-*g*-PVA/Lin/D_2O 10/15/75 wt%	0.13 ± 0.01	29 ± 3 g/L

7. References

(1) Bartolini, A.; Tempesti, P.; Resta, C.; Berti, D.; Smets, J.; Aouad, Y. G.; Baglioni, P. Poly(Ethylene Glycol)-Graft-Poly(Vinyl Acetate) Single-Chain Nanoparticles for the Encapsulation of Small Molecules. *Physical Chemistry Chemical Physics* **2017**, *19* (6), 4553–4559. https://doi.org/10.1039/C6CP07967A.

(2) Bartolini, A.; Tempesti, P.; Ghobadi, A. F.; Berti, D.; Smets, J.; Aouad, Y. G.; Baglioni, P. Liquid-Liquid Phase Separation of Polymeric Microdomains with Tunable Inner Morphology: Mechanistic Insights and Applications. *Journal of Colloid and Interface Science* **2019**, *556*, 74–82. https://doi.org/10.1016/j.jcis.2019.08.015.

(3) Hansen, C. M. *Hansen Solubility Parameters: A User's Handbook.*; 2007.

(4) Montis, C.; Maiolo, D.; Alessandri, I.; Bergese, P.; Berti, D. Interaction of Nanoparticles with Lipid Membranes: A Multiscale Perspective. *Nanoscale* **2014**, *6* (12), 6452–6457. https://doi.org/10.1039/C4NR00838C.

(5) Schneider, C. A.; Rasband, W. S.; Eliceiri, K. W. NIH Image to ImageJ: 25 Years of Image Analysis. *Nat Methods* **2012**, *9* (7), 671–675. https://doi.org/10.1038/nmeth.2089.

(6) Zhang, F.; Ilavsky, J.; Long, G.G.; Quintana, J.P.G.; Allen, A.J.; Jemian, P.R. Glassy carbon as an absolute intensity calibration standard for small-angle scattering. *Metallurgical and Materials Transactions A* **2010**, 41, 1151–1158.

(7) Guinier, A.; Fournet, G.; Walker, C. B. *Small Angle Scattering of X-Rays*, J. Wiley&Sons, New York.; 1955.
